# Pancreas Whole Tissue Transcriptomics Highlights the Role of the Exocrine Pancreas in Patients With Recently Diagnosed Type 1 Diabetes

**DOI:** 10.3389/fendo.2022.861985

**Published:** 2022-04-13

**Authors:** Tommi Välikangas, Niina Lietzén, Maria K. Jaakkola, Lars Krogvold, Morten C. Eike, Henna Kallionpää, Soile Tuomela, Clayton Mathews, Ivan C. Gerling, Sami Oikarinen, Heikki Hyöty, Knut Dahl-Jorgensen, Laura L. Elo, Riitta Lahesmaa

**Affiliations:** ^1^Turku Bioscience Centre, University of Turku and Åbo Akademi University, Turku, Finland; ^2^InFLAMES Research Flagship Center, University of Turku, Turku, Finland; ^3^Department of Mathematics and Statistics, University of Turku, Turku, Finland; ^4^Pediatric Department, Oslo University Hospital, Oslo, Norway; ^5^Institute of Clinical Dentistry, Faculty of Dentistry, University of Oslo, Oslo, Norway; ^6^Department of Medical Genetics, Oslo University Hospital, Oslo, Norway; ^7^Department of Pathology, University of Florida, Gainesville, FL, United States; ^8^Department of Medicine, University of Tennessee Health Science Center, Memphis, TN, United States; ^9^Faculty of Medicine and Health Technology, Tampere University, Tampere, Finland; ^10^Fimlab Laboratories, Pirkanmaa Hospital District, Tampere, Finland; ^11^Institute of Clinical Medicine, Faculty of Medicine, University of Oslo, Oslo, Norway; ^12^Institute of Biomedicine, University of Turku, Turku, Finland

**Keywords:** DiViD, exocrine pancreas, gene expression, pancreatic islet, pancreas, transcriptomics, type 1 diabetes

## Abstract

Although type 1 diabetes (T1D) is primarily a disease of the pancreatic beta-cells, understanding of the disease-associated alterations in the whole pancreas could be important for the improved treatment or the prevention of the disease. We have characterized the whole-pancreas gene expression of patients with recently diagnosed T1D from the Diabetes Virus Detection (DiViD) study and non-diabetic controls. Furthermore, another parallel dataset of the whole pancreas and an additional dataset from the laser-captured pancreatic islets of the DiViD patients and non-diabetic organ donors were analyzed together with the original dataset to confirm the results and to get further insights into the potential disease-associated differences between the exocrine and the endocrine pancreas. First, higher expression of the core acinar cell genes, encoding for digestive enzymes, was detected in the whole pancreas of the DiViD patients when compared to non-diabetic controls. Second, In the pancreatic islets, upregulation of immune and inflammation related genes was observed in the DiViD patients when compared to non-diabetic controls, in line with earlier publications, while an opposite trend was observed for several immune and inflammation related genes at the whole pancreas tissue level. Third, strong downregulation of the regenerating gene family (*REG*) genes, linked to pancreatic islet growth and regeneration, was observed in the exocrine acinar cell dominated whole-pancreas data of the DiViD patients when compared with the non-diabetic controls. Fourth, analysis of unique features in the transcriptomes of each DiViD patient compared with the other DiViD patients, revealed elevated expression of central antiviral immune response genes in the whole-pancreas samples, but not in the pancreatic islets, of one DiViD patient. This difference in the extent of antiviral gene expression suggests different statuses of infection in the pancreas at the time of sampling between the DiViD patients, who were all enterovirus *VP1*+ in the islets by immunohistochemistry based on earlier studies. The observed features, indicating differences in the function, status and interplay between the exocrine and the endocrine pancreas of recent onset T1D patients, highlight the importance of studying both compartments for better understanding of the molecular mechanisms of T1D.

## Introduction

Type 1 diabetes (T1D) is an autoimmune disease characterized by the progressing destruction of pancreatic β-cells and the related loss of insulin production, blood glucose control and finally extreme hyperglycemia and ketoacidosis in untreated patients (1–3). The exact cause of T1D remains unknown with both genetic (4, 5) and environmental factors suspected to play a role in the disease etiology. The disease progression is assumed to be initiated by inflammation of the pancreas and the islets of Langerhans (pancreatic islets) containing β-cells.

In the Diabetes Virus Detection (DiViD) study, samples from the pancreatic tissue of six living adults with recently diagnosed T1D were collected using pancreatic tail resection by laparoscopy (6). The insulin secretion capability of the isolated pancreatic islets was shown to be recoverable when removed from the diabetic environment, and all transcripts in the human insulin pathway were present, but less expressed in the DiViD patients when compared to the nondiabetic controls (7). To investigate the potential link between virus infections and T1D, indicators of virus infection in the samples have been explored with multiple approaches, and the presence of enteroviruses has been shown in the pancreatic islets of all six DiViD patients (8).

T1D is a disease of the whole pancreas, not just the pancreatic islets, although most of the studies into the etiology of T1D have focused on the endocrine tissue of the pancreas. The pancreas of individuals with T1D have been observed to be smaller in size compared to the pancreases of patients with type 2 diabetes (T2D) or control subjects (9, 10). In addition to decreased volume, other anomalies, including various histological changes in the exocrine pancreas of diabetic patients, have been discovered (9). Also pancreatic exocrine dysfunction or pancreatic insufficiency are frequent for T1D patients (9, 11). Furthermore, preserved beta cell insulin secretion has been connected with preserved acinar cell function and volume (9, 12). Finally, infiltration of the exocrine pancreas by immune cells in connection with T1D has been noticed in several studies (10, 13).

There has been an increasing body of evidence linking viral infections to the occurrence or facilitation of T1D (14). While multiple types of different viruses have been associated with T1D (15, 16), the common enteroviruses, especially the group B Coxsackieviruses, have been proposed as strong candidates for promoting T1D (17, 18). Enteroviruses possess a strong affinity for the pancreatic islets (19), and the presence of low grade enterovirus infection based on the detection of enterovirus capsid protein *VP1* in the pancreatic islets has been demonstrated in several studies, including the DiViD study (18–20). Compared to an acute infection, in persistent infection the virus may be present at extremely low numbers and the replication of the virus might be decreased, rendering the virus difficult to detect (20). Recently, studies utilizing highly sensitive fluorescent *in situ* hybridization method to detect enteroviral RNA in pancreas tissue samples, have shown the presence of the virus both in the islets and in the exocrine pancreas of T1D patients and at-risk individuals positive for T1D autoantibodies (21, 22). The progression of enterovirus infection and the possible facilitation of T1D by the infection are likely influenced by the virus itself, as well as host responses and host factors (e.g. gender, age, dietary factors, infection timing, genetics) (20). Generally, viral agents have been observed to be able to regulate host responses, and to downregulate the inflammatory responses in human cells (23). There are also clear differences in the immunogenicity of different viruses, including different CVB1 strains (24–26). In addition, several cell surface receptors for enteroviruses are recognized, and the receptor usage seems to differ to some extent between distinct members of the virus family (19). This will likely result in differences in the cell populations that are most susceptible for infection as well as the outcomes of infection.

To investigate the role of both the exocrine and the endocrine pancreas in early T1D, we characterized total gene expression in the whole pancreas tissue of the DiViD study patients with recently diagnosed T1D and compared it with the pancreas tissue transcriptomes of non-diabetic organ donors. To further characterize the contribution of different pancreas compartments on the observed changes, gene expression in the whole pancreas was compared to the gene expression in the pancreatic islets of the DiViD patients. Additionally, a gene expression dataset produced by an independent laboratory from pancreas whole-tissue samples of the same DiViD patients was used to verify part of the results. The combined analysis of these three gene expression datasets allowed for a comprehensive exploration of gene expression and systemic changes in the pancreas related to recently diagnosed T1D. Finally, in addition to investigating gene expression in the exocrine and endocrine pancreas, the generated datasets also enabled the exploration of potential enterovirus related signals in the whole pancreas and in the pancreatic islets of the DiViD patients.

## Materials and Methods

Pancreas tissue samples from recent onset T1D patients participating in the Diabetes Virus Detection (DiViD) study (6) were analyzed. The samples were collected by pancreatic tail resections performed 3–9 weeks after the diagnosis of T1D. The DiViD study was approved by the Norwegian Government´s Regional Ethics Committee, and written informed consent was obtained from all patients.

### Whole Pancreas Transcriptome 1

RNA was isolated from pancreas whole tissue samples preserved in RNAlater, using RNeasy Plus Mini kit (Qiagen). Samples from the DiViD cases 2-6, (two females and three males, aged 24-35 years) (6) were prepared and analyzed. Based on the quality control results, RNA from the pancreas tissue sample of DiViD case 2 was degraded (**Supplementary Figure 1**), and therefore an adjusted protocol was used for RNA-sequencing analysis of this sample. The adjusted protocol for DiViD case 2 was provided by Illumina TruSeq Stranded Total RNA Reference Guide (Document # 1000000040499 v00), where during library preparation of the sample, the RNA fragmentation time was reduced from 8 min to 6 min. Three commercial human pancreas total RNA preparates were used as controls (#AM7954, Ambion; #540023, Agilent Technologies; #636577, Clontech) (**Supplementary Table 1**). RNA-sequencing was performed using Illumina TruSeq^®^ Stranded total RNA protocol and Illumina HiSeq 3000 instrument with TruSeq v4 sequencing. Paired-end sequencing with 2 x 150 bp read length with 6 bp index run was used.

### Whole Pancreas Transcriptome 2

Independent total RNA-sequencing was performed for separate pancreas whole tissue pieces from the DiViD patients (cases 1-6) in the Oslo University Hospital (8). The sequencing was done using Illumina TruSeq^®^ Stranded total RNA protocol and Illumina HiSeq 2000 instrument with paired-end sequencing and 2 x 100 bp read length.

### Pancreatic Islet Transcriptome

The laser-capture microarray data was generated for the DiViD cases 2-6 and for 18 Network for Pancreatic Organ donors with Diabetes (nPOD) non-diabetic controls and normalized as described before (27).pt?>

### Data Analyses

Cell type proportions in the whole pancreas transcriptome 1 dataset were estimated using the online deconvolution tool CIBERSORT (28) with a signature matrix constructed from pancreatic single-cell data (29) under the accession ID E-MTAB-5061 in ArrayExpress. The utilized signature matrix is available as **Supplementary Data 1**. For exploring differential expression between the DiViD cases and the controls, the reproducibility optimized test statistic (ROTS) (30), shown to perform well with RNA-Seq data (31), was used. ROTS false discovery rate (FDR) 0.05 and a fold change (FC) threshold of two were used to define the differentially expressed (DE) genes. To explore the enrichment of gene ontology (GO) biological processes (BP) among the DE genes, the Database for Annotation, Visualization and Integrated Discovery (DAVID) (32) version 6.8 was used. The GO FAT terms were considered, filtering out the broadest GO term annotation categories. Only GO BP terms with FDR ≤ 0.05 were considered as enriched. Protein-protein interactions among the gene products of interest were queried from the STRING functional protein association networks database (33) using both the predicted and known interactions. Only high confidence interactions (confidence score ≥ 0.7) were considered. To compare gene expression between the DiViD cases, gene expression in each dataset was z-score transformed using the case samples in each dataset only.

To ensure comparability, all datasets were filtered and preprocessed as similarly as possible. For details related to preprocessing and data analysis of the datasets, see **Supplementary Methods**.

## Results

### Recent Onset T1D Associated Gene Expression Profile Can be Identified in the Whole Pancreas Tissue

**Figure 1:**
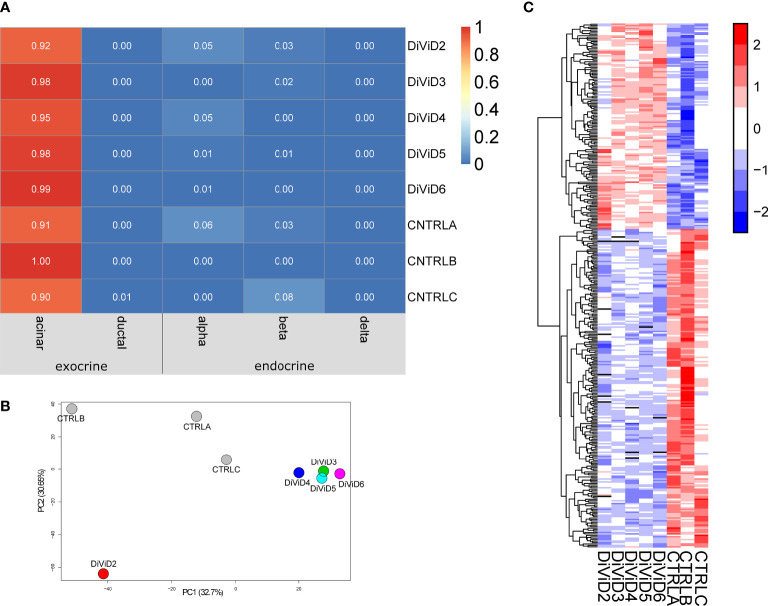
The whole pancreas tissue transcriptome 1. **(A)** Estimated cell type proportions in the pancreatic tissue samples. The online deconvolution tool CIBERSORT (28) was utilized to obtain the estimates. **(B)** Samples of the data along the first two principal components. **(C)** The z-score standardized expression of the differentially expressed genes in the data. The differentially expressed genes were identified using the reproducibility optimized test statistic (ROTS) (30) with a false discovery rate of ≤ 0.05 and a fold change of ≥ 2. In all panels, CTRLA refers to the Agilent commercial control (non-diabetic organ donor), CTRLB refers to the Ambion commercial control (non-diabetic organ donor) and CTRLC refers to the commercial Clontech control (non-diabetic organ donor).

We performed RNA-sequencing for pancreas tissue samples collected from five recent onset T1D patients, as well as for three commercial human pancreas tissue RNA preparates of non-diabetic adults. To get a rough estimation on the contribution of different pancreatic cell types on total tissue transcriptomes of each sample, cell type deconvolution was performed using the CIBERSORT tool (28). Acinar cells are clearly the most abundant cell type in the pancreas, which was also reflected in the cell type deconvolution results of the current RNA-sequencing data. While the contribution of acinar cells on the RNA-sequencing data was estimated to be over 90%, the estimated contribution of endocrine cells was only few percent for all the samples with no difference in proportions between the cases and the controls. (**Figure 1A**)

The pancreas tissue transcriptomes of individuals with recent onset of T1D were clearly separated from the control samples on the first two principal components of the Principal Component Analysis (PCA) plot (**Figure 1B**). Furthermore, the differential expression analysis of the transcriptomics data revealed T1D-associated differences in the expression of 365 genes (**Figure 1C** and **Supplementary Table 2**).

### T1D Influences the Expression of Genes in the Exocrine and the Endocrine Pancreas

**Figure 2:**
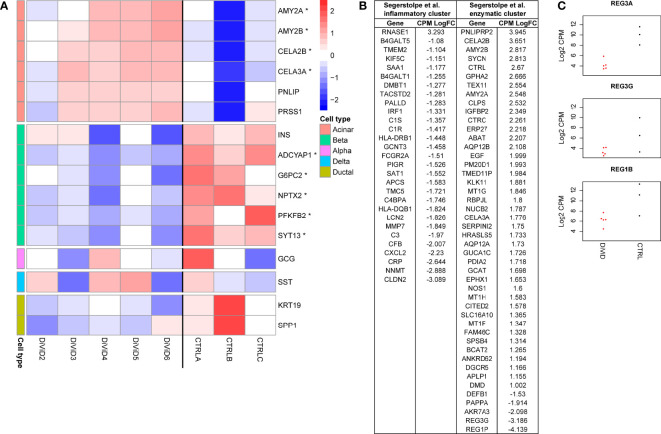
**(A)** Z-score normalized expression of marker genes, and selected genes enriched in the different pancreas cell types (29, 34) that are also differentially expressed (* false discovery rate of ≤ 0.05 and a fold change of ≥ 2) in the whole pancreas transcriptome 1. CTRLA refers to the Agilent commercial control (non-diabetic organ donor), CTRLB refers to the Ambion commercial control (non-diabetic organ donor) and CTRLC refers to the commercial Clontech control (non-diabetic organ donor). **(B)** Differentially expressed genes in the whole pancreas transcriptome 1 associated with the two different acinar cell subpopulations suggested by Segerstolpe et al. (29). **(C)** The log_2_ transformed counts per million (CPM) expression of three Reg-family genes in the whole pancreas transcriptome 1. Red color = samples from the DiViD patients, black color = samples from non-diabetic organ donors.

Study on the cell type enriched genes in the pancreas showed concurrent T1D associated differences in the beta-cell and acinar-cell gene expression (**Figure 2A**). Also for markers of the pancreatic ductal cells, *KRT19* and *SPP1*, a trend towards higher expression in the control samples was observed, but this difference was not statistically significant. No T1D associated differences were observed in the expression of the alpha-cell and delta-cell markers *GCG* and *SST*, respectively.

The loss of insulin-producing beta-cells in the pancreatic islets is the core pathogenic feature in T1D. Also in the current study, *INS* mRNA levels were lower in every tissue sample from individuals with recent onset T1D when compared with the control samples, although the statistical significance remained slightly above the used FDR threshold of 0.05 (**Figure 2A**). Furthermore, five other genes shown to be enriched in the pancreatic beta cells by single cell RNA-sequencing (*ADCYAP1, G6PC2, NPTX2, PFKFB2, SYT13*) (29, 34) were significantly downregulated in the tissue samples of individuals with recent onset T1D. In addition to impaired insulin production, these results show a broader reduction in pancreatic beta-cell functions shortly after the diagnosis of T1D.

The core function of the acinar cells of the exocrine pancreas is the production of digestive enzymes. Transcripts encoding for several digestive enzymes including *AMY2A, AMY2B, CELA3A* and *CELA2B* were significantly upregulated in the pancreas tissue samples of T1D patients when compared with the control samples (**Figure 2A**). A similar trend, although not statistically significant, was also observed for *PNLIP* and *PRSS1* often used as markers of acinar cells. Segerstolpe et al. have utilized single cell RNA-sequencing data to further define two distinct acinar cell subpopulations: the inflammatory cluster characterized by the increased expression of inflammatory genes and the enzymatic cluster characterized by the elevated levels of key acinar genes encoding for digestive enzymes (29). Interestingly, many of the differentially expressed genes between the T1D and control pancreatic samples in the current study were also distinguishing the suggested acinar cell subpopulations from each other. Among the overlapping genes, almost all enzymatic cluster associated genes were upregulated in the pancreas tissue samples of recent onset T1D patients. On the other hand, the overlapping genes associated with the inflammatory acinar cell cluster were downregulated in the pancreas tissue samples of recent onset T1D patients, potentially reflecting a T1D associated change in balance between the different acinar cell subpopulations (**Figure 2B**). Finally, *REG3A* has been shown to mark a subpopulation of acinar cells located close to the islets of Langerhans (34), and it has been suggested to support pancreatic islet function (35). Interestingly, *REG3A* was the most strongly downregulated gene in the pancreas tissue samples of individuals with recent onset T1D (**Figure 2C** and **Supplementary Table 2**). Additionally *REG1B* and *REG3G*, also members of the *Reg*-family genes, were clearly downregulated in the pancreas tissue samples of individuals with recent onset T1D (**Figure 2C**).

### Inflammation and Immune Response Associated Genes Are Downregulated in the Pancreas Tissue of Recent Onset T1D Patients

Based on the functional gene ontology (GO) enrichment analysis of the differentially expressed genes, significant T1D associated differences were observed in the expression of genes associated with extracellular matrix organization, cell migration and cell adhesion (**Supplementary Figure 2**), potentially reflecting changes in cell-cell contacts and communication. These changes might also influence the ability of enteroviruses and other viruses to enter the cells.

**Figure 3:**
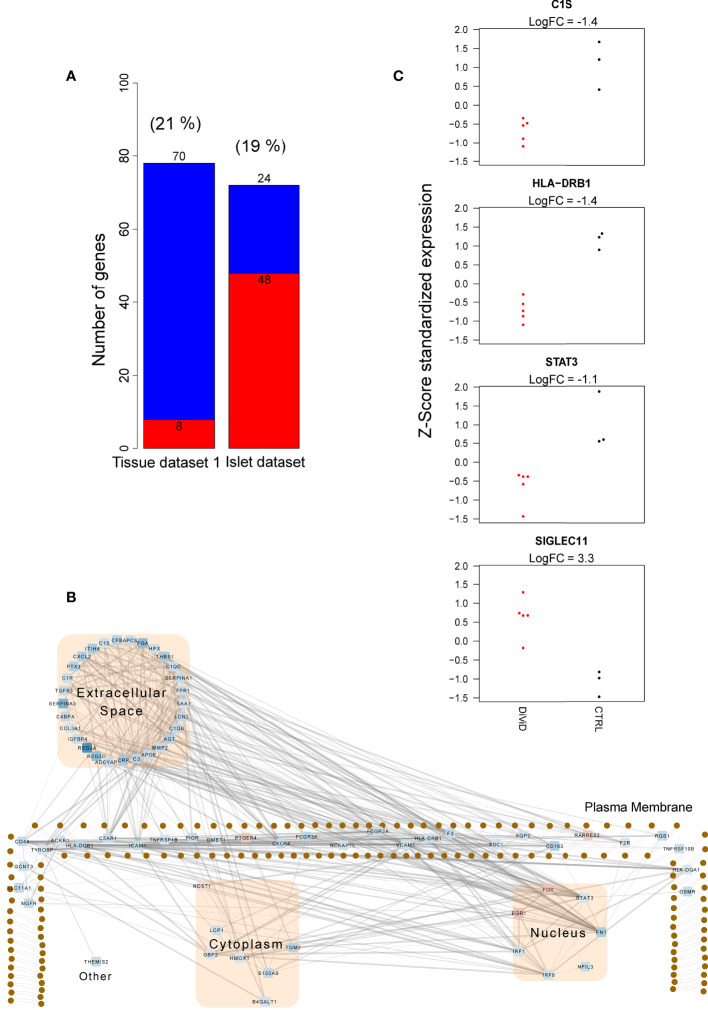
Immune and inflammatory responses in the whole pancreas transcriptome 1 and the pancreatic islet transcriptome. **(A)** The number of up-regulated (red) and down-regulated (blue) immune and inflammatory response genes between the DiViD patients and the controls. The differentially expressed (DE) genes were identified using the reproducibility optimized test statistic (ROTS) (30) with a false discovery rate of ≤ 0.05 and |FC| ≥ 2. Genes in the biological processes GO:0006955 (immune response) and GO:0006954 (inflammatory response) were examined. The proportion of DE genes in each dataset mapping to the examined gene ontology (GO) terms is shown in brackets. The difference in the number of both up- and down-regulated genes of all the included immune and inflammatory response genes between the whole tissue transcriptome 1 and the islet transcriptome was highly statistically significant (P<0.0001, two proportions z-test). **(B)** The subcellular protein-protein interaction network of the products of the differentially expressed genes in the biological processes GO:0006955 (immune response) and GO:0006954 (inflammatory response) in the whole tissue transcriptome 1. The protein-protein interactions were queried from the STRING functional protein association database (33). Only high confidence interactions (confidence score ≥ 0.7) were regarded. Ingenuity Pathway Analysis (IPA) (QIAGEN Inc.) (36) was applied for the determination of subcellular locations and Cytoscape (37) for the visualization of the network. The color of the nodes in the network represent the expression of the genes: red represents up-regulation, while blue represents down-regulation. **(C)** Z-score standardized expression of the selected differentially expressed inflammatory genes in the whole tissue transcriptome 1. Red color = samples from the DiViD patients, black color = samples from non-diabetic organ donors.

In addition, several genes associated with inflammation and immune responses were differentially expressed in the pancreas tissue samples and pancreatic islets between the DiViD patients and non-diabetic controls (**Figure 3A** and **Supplementary Figure 2**, **Supplementary Table 3**). Interestingly, these differentially expressed genes were almost exclusive for one tissue type. In line with earlier studies (38), the upregulation of HLA class I genes and several interferon response genes was observed in the pancreatic islets of the DiViD patients when compared with non-diabetic controls. On the other hand, in the pancreas whole-tissue samples, several genes involved in the early steps of complement activation (e.g. *C1qB, C1qC, C1R, C1S, C3*), Fc gamma receptors (*FCGR2A, FCGR3A*), and Tumor necrosis factor receptors (*TNFRSF1B, TNFRSF10B, TNFRSF12a*) were significantly downregulated in the DiViD patients when compared with non-diabetic controls (**Figures 3B, C**). Some of these downregulated immune and inflammatory genes, including *LCN2, CLDN2*, MHC class II genes (*HLA-DQB1* and *HLA-DRB1*) and some complement genes, were also among the genes distinguishing the inflammatory acinar cell cluster from the enzymatic acinar cell cluster in (29), supporting the potential contribution of the exocrine pancreas on the changes observed in the pancreas total tissue samples. Also, three transcription factors with central roles in the activation of immune and inflammatory responses (*IRF1, IRF8* and *STAT3*) were significantly downregulated in the whole pancreas of T1D patients. Intriguingly, the most strongly upregulated gene in the pancreas tissue samples of recent onset T1D patients was *SIGLEC11* (**Figure 3C**), which is an immune inhibitory *SIGLEC* receptor.

### Antiviral Immune Response Signature in the Exocrine Pancreas of One T1D Patient

**Figure 4:**
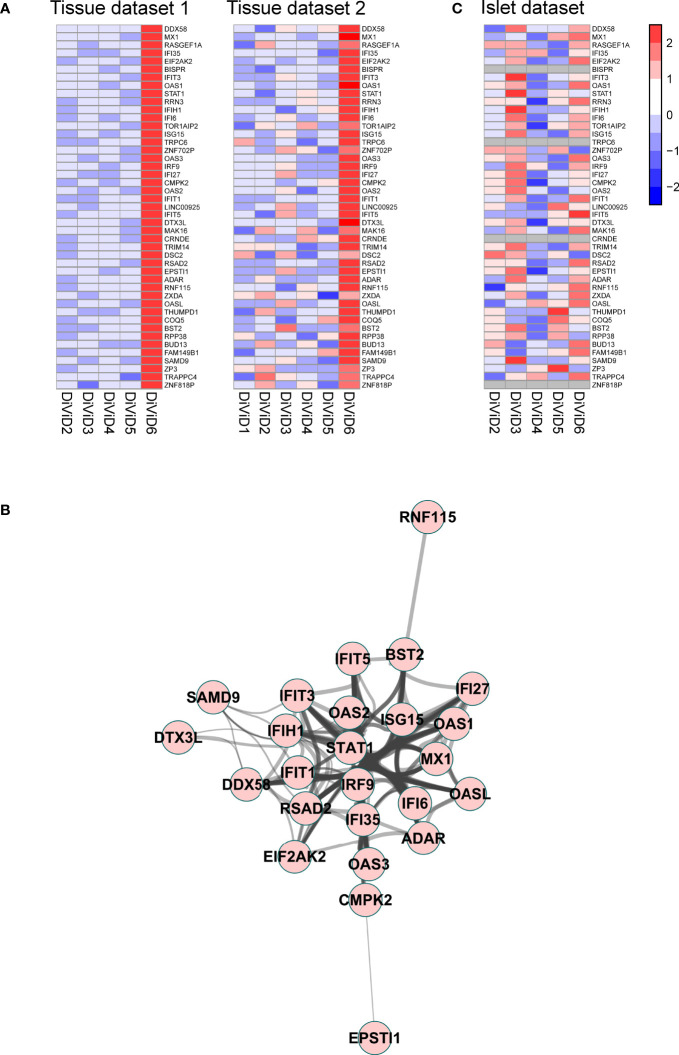
**(A)** A signature of 45 genes was discovered differentiating the DiViD case 6 from the other DiViD patients. Z-score standardized expression of these genes is shown in two independent pancreas total tissue RNA-sequencing datasets from the DiViD patients. **(B)** Protein-protein interaction network within the signature genes shows a cluster of genes with central roles in antiviral immune responses. The protein-protein interactions were queried from the STRING functional protein association database (33, 39) and visualized with the Cytoscape software (37). Only high confidence interactions (combined interaction score ≥ 0.7) were regarded. **(C)** Z-score standardized expression of the 45 signature genes in the pancreatic islet transcriptome from the DiViD patients.

In addition to the T1D associated differences in gene expression in the pancreas, our aim was to understand the differences between the individual recent onset T1D patients in the current study. For that, we studied the top 100 most highly expressed transcripts in each DiViD case relative to any other DiViD case, and named these gene sets as individual signatures. As further validation, similar analysis was performed on an independent RNA-sequencing dataset from distinct pancreas tissue samples collected at the same time from the same individuals. Genes that were included in the individual signatures in both datasets were considered as reliable observations. An individual signature of 45 genes was discovered in both RNA-sequencing datasets for DiViD case 6 (**Figure 4A**). Only in this signature of DiViD case 6, strong known interactions between the signature transcripts were detected (**Figure 4B**), indicating a presence of a group of tightly related features. This signature included several interconnected transcripts with central roles in antiviral interferon responses, including *EIF2AK2, MX1, STAT1* and *ISG15* (**Figure 4B**), suggesting the presence of viral infection in the pancreas of DiViD case 6. Interestingly, when these results were compared with microarray transcriptomics data collected from the laser capture microdissected islets of the same individuals, no such clear trend was observed (**Figure 4C**). Furthermore, the expression of the antiviral signature genes in DiViD case 6 was clearly higher than in the control pancreas tissue samples (**Supplementary Figure 3**). In conclusion, our data indicates strong contribution from the exocrine pancreas, rather than the pancreatic islets, on the antiviral signature observed in the current study, which is specific for DiViD case 6.

## Discussion

Although T1D is primarily a disease of the pancreatic beta-cells, understanding of the disease-associated alterations in the whole pancreas could be important for the improved treatment or prevention of the disease. In this study, our aim was to characterize changes in pancreas gene expression that are associated with the early stages of human T1D soon after the diagnosis of the disease. Our pancreas tissue RNA-sequencing results showed concurrent T1D associated differences in the expression of both beta-cell and acinar cell linked transcripts.

T1D has been associated with reduced pancreas size (9, 10, 40) and exocrine dysfunction [summarized in (9)]. The reduction in pancreas weight and volume in T1D could occur even before beta-cell loss and disease diagnosis (40, 41), and might be one of the early T1D predisposing changes in the pancreas. Curiously, recent studies have shown preserved acinar cell size (42) and density (43) in the pancreases of donors with T1D, implying that the reduced pancreas volume in T1D could be due to a reduced number of acinar cells rather than acinar atrophy. Interestingly, in the current study, higher expression of core acinar cell genes encoding for digestive enzymes was observed in the pancreas tissue samples from the recent onset T1D patients than from the controls. Increased expression of genes encoding for digestive enzymes might actually reflect an attempt of the remaining acinar cells to compensate for the reduced functional capacity associated with a reduced pancreas volume, and could even be linked to a shift in the balance between different acinar cell subpopulations, as suggested by Segerstolpe et al. (29). Although our cell type deconvolution analysis did not reveal any major differences in the proportion of acinar cells between the DiViD patient samples and the controls, further studies at single cell level would be required to confirm this as the accuracy of computational deconvolution is lower than that of the corresponding experimental approaches (44).

In addition to the subpopulation of acinar cells expressing high levels of genes encoding for secretory digestive enzymes, Segerstolpe et al. suggested the presence of another acinar cell subpopulation characterized by the elevated expression levels of inflammatory and immune associated genes (29). Interestingly, a clear observation in the current pancreas tissue RNA-sequencing data was the downregulation of several of these genes, including *LCN2, CLDN2* and MHC class II genes, in the pancreas of the recent onset T1D patients. The potentially reduced numbers of acinar cells expressing inflammation and immune regulatory genes could early on contribute to making the exocrine pancreas less capable of responding to immune challenges, such as virus infections. Still, the regulation of immune and inflammatory processes occurs at multiple levels, and other factors are also likely to influence this. For example, the inhibitory *SIGLEC* receptors dampen the inflammatory and immune responses, but they are also thought to have a role in the maintenance of self-tolerance (45). The inhibitory *SIGLEC* receptor, *SIGLEC11*, known to be expressed in the pancreas (46), was the most strongly downregulated gene in the pancreas of the recent onset T1D patients, and in earlier studies it has been associated with neuroinflammation (45, 46). Interestingly, while downregulated in the whole pancreas, SIGLEC11 was not detected as significantly regulated in the pancreatic islets of the DiViD patients when compared to non-diabetic controls. Moreover, the downregulation of immune and inflammatory genes in the whole pancreas differs from earlier reports from the pancreatic islets and beta-cells, showing T1D associated upregulation of such genes, including for example MHC class I and II genes, and interferon response related *STAT1* and *MX1* in (38, 47). This suggests largely differing expression patterns for immune and inflammatory genes between the endocrine and the exocrine pancreas. However, although the global expression of immune and inflammatory genes was downregulated in the pancreas tissue samples of the individuals with recent onset T1D in the current data, local infiltration of immune cells and activation of immune responses might still be present also in these samples.

The interplay between the exocrine and the endocrine pancreas is thought to have an important role in maintaining pancreatic homeostasis (35). *Reg*-family genes are thought to promote pancreatic islet growth and regeneration (48), and *REG3A* was discovered as a marker of acinar cells close to the pancreatic islets (34). Strong downregulation of *REG3A*, and also *REG3G* and *REG1B* in the pancreas of T1D patients in the current study could therefore reflect impaired interplay between the exocrine and the endocrine pancreas. Furthermore, recent reports on intermediate cell types containing simultaneously both exocrine and endocrine features (49) or mixed characteristics of different endocrine cell types (50), further complicate the classification and functional understanding of distinct cell types. Therefore, broader studies at the level of single cells are required to understand the presence and relationships between the different cell subtypes in the pancreas in T1D. However, in addition to being related to pancreatic islet growth, regeneration and the acinar cell type, members of the third subclass of the *Reg* family, have been observed to be associated with host defenses and hyperglycemia and it has been suggested that bacteria, injury, interleukins (51) or hyperglycemic conditions (52) can regulate or inhibit the expression of *REG3A*, further complicating the picture for the possible underlying cause for the observed gene expression.

Enteroviruses are thought to be one of the potential triggers for the development of T1D. Several studies have shown the presence of enteroviruses in the pancreatic beta-cells of T1D patients more often than in control populations (8, 53, 54). In addition, recent studies have shown the detection of enterovirus RNA also in the exocrine pancreas of T1D and non-diabetic individuals (21, 22). Krogvold et al. explored the presence of enteroviruses in the pancreas of the DiViD patients using immunohistochemistry (IHC), reverse transcription polymerase chain reaction (RT-PCR) and RNA-sequencing (8). While no viral genome was detected in the pancreas tissue samples using RNA-sequencing, sensitive RT-PCR detected enterovirus RNA in the pancreas tissue in one of them, the DiViD case 6. On the other hand, pancreatic islets were positive for the enterovirus *VP1* protein in IHC in all the six DiViD cases and culture supernatants of isolated islets were positive for enterovirus RNA by RT-PCR in the DiViD cases 2, 4, 5 and 6. Finally, an earlier study showed overexpression of several interferon stimulated genes in the insulitic islets of the DiViD patients, when compared to non-insulitic islets from organ donors without diabetes (38). Our results, dominated by the exocrine pancreas, showed clearly higher expression of central antiviral immune response genes in the pancreas tissue sample of DiViD case 6 compared to all the other DiViD cases, and are thereby well in line with the earlier data on the presence of enteroviruses (8). Many of the antiviral immune response genes upregulated in the pancreas tissue sample of DiViD case 6 were also included in an enterovirus induced blood gene expression signature defined in our earlier study (55), supporting the link to enterovirus infection. On the other hand, studying the same set of central antiviral immune response genes in microarray data collected from the pancreatic islets of the same individuals showed no clear differences between the DiViD cases, indicating similar infection status in their pancreatic islets. These results highlight the importance of studying both the exocrine and the endocrine pancreas at an individual level when aiming to understand the role of enteroviruses in the development of T1D.

In our whole pancreas transcriptome 1, clear downregulation of genes and processes related to extracellular matrix organization, cell migration, cell adhesion, cell motility and cell surface receptors was observed (**Supplementary Figure 2**). Similar downregulation of genes and processes associated with cell-cell contacts, extracellular matrix and cell surface receptors was observed in a recent study by us and others as a result of a carrier-state-type persistent infection by two Group B coxsackievirus (CVB) strains in a pancreatic cell line (26). Moreover, many cell surface receptors are known for enteroviruses including members of the integrin and immunoglobulin superfamilies typically involved in cell adhesion (19). In earlier studies over two decades ago, it was observed that the human enterovirus coxsackievirus A21 binds with high affinity to the intercellular adhesion molecule 1 (ICAM1) (56, 57) for cell entry, a phenomenon already known earlier in relation to several rhinoviruses. Persistent infection by coxsackie B viruses of human microvascular endothelial cells have been observed to result in increased expression of adhesion molecules, including ICAM1, especially shortly after the infection (<30-40 days after infection) (58), fluctuating to lower levels afterwards. More generally, the interplay between viral proteins and host proteins, namely cellular receptors and proteins, largely determines the attachment, entry and internalization of the virus into the host cells (59). Interestingly, among other cell adhesion molecules, ICAM1 was also observed to be downregulated in the recently diagnosed DiViD patients when compared to the non-diabetic controls in our whole pancreas data (**Supplementary Table 2**). Such downregulation of cell adhesion and cell surface receptors observed in this study and as a response to persistent carrier state CVB infection by (26), can reflect the hosts attempt in controlling the infection after a prolonged infectious state. However, even though there is a large amount of evidence linking enteroviruses and viruses in general to cell surface molecules related to cell adhesion, the exact mechanisms of virus-host interplay regarding these molecules are unknown and require further research.

Due to the invasiveness of pancreas tissue sampling, there are clear limitations in accessing appropriate control samples for the unique DiViD study of living young adults with recently diagnosed T1D. In the current study, three commercial pancreas total tissue controls from different sources and with differing cadaveric donor characteristics were used with an aim to minimize systematic bias due to the limitations with the control samples. Overall, our data suggests that the development of T1D is associated with co-occurring changes in the exocrine and the endocrine pancreas, and might involve an imbalance and impaired communication between different cell populations in the pancreas. In the future, broader comparisons across different levels of data from deep bulk omics analyses to single cell profiling and imaging analyses with spatial information could provide valuable knowledge for deeper understanding on the pathogenesis of T1D. Such cross-platform studies could also better take into account the heterogeneity of pancreas tissue and its effects on the unique readouts of each platform.

## Data Availability Statement

The datasets presented in this article are not readily available because of the sensitive nature of the data and possible high risks associated with patient confidentiality. Requests to access the datasets should be directed to knut.dahl-jorgensen@medisin.uio.no.

## Ethics Statement

DiViD study was approved by the Norwegian Government´s Regional Ethics Committee, and written informed consent was obtained from all patients. The patients/participants provided their written informed consent to participate in this study.

## Author Contributions

TV participated in designing the study, drafted the manuscript with NL and MJ, processed all the datasets included in the study, performed the data analysis and interpreted the results. NL participated in designing the study, drafted the manuscript with TV and MJ, analyzed the data and interpreted the results. MJ participated in drafting the manuscript with TV and NL and performed the cell type deconvolution analysis. LK was responsible for clinical coordination, the recruitment of patients, data collection, the whole transcriptome sequencing of the whole tissue transcriptome 2 together with ME and KD-J and participated in interpretation of the results. ME was responsible for the whole transcriptome sequencing of the whole tissue transcriptome 2 together with LK and KD-J. HK and ST participated in designing the study, performed the whole transcriptome sequencing analysis of the whole tissue transcriptome 1 and participated in interpreting the results. CM was responsible for the analysis of the islet transcriptome data together with IG. IG was responsible for generating the islet transcriptome data together with CM and participated in interpreting the results. SO was responsible for generating the pancreatic tissue samples for the whole tissue transcriptome 1 together with HH. HH was responsible for generating the pancreatic tissue samples for the whole tissue transcriptome 1 together with SO and participated in interpreting the results. KD-J had the initial idea and design of the DiViD study, participated in study design, funding, regulatory issues, international collaboration, data collection and interpreting the results. LE was responsible for the study design, supervised the performed data analysis, and participated in interpreting the results and drafting the manuscript. RL was responsible for the study design, supervised the study, and participated in interpreting the results and drafting the manuscript. All authors contributed to the article and approved the submitted version.

## Funding

The DiViD study was funded by South-Eastern Norway Regional Health Authority (Grant to KD-J), The Novo Nordisk Foundation (Grant to KD-J) and through PEVNET. The present work was financially supported by the European Commission (Persistent Virus Infection in Diabetes Network [PEVNET] Frame Programme 7, contract number 261441). RL and LE groups are also supported by InFLAMES Flagship Programme of the Academy of Finland (decision number: 337530). NL was supported by the Academy of Finland decision no. 287423. RL received funding from the Academy of Finland (grants 292335, 294337, 319280, 31444, 319280, 329277, 331790), Business Finland and by grants from the JDRF, the Sigrid Jusélius Foundation (SJF), Jane and Aatos Erkko Foundation, the Novo Nordisk Foundation, Finnish Diabetes Foundation and the Finnish Cancer Foundation. LE reports grants from the European Research Council ERC (677943), European Union’s Horizon 2020 research and innovation programme (955321), Academy of Finland (296801, 310561, 314443, 329278, 335434 and 335611), and Sigrid Juselius Foundation, during the conduct of the study. CM and IG were supported by National Institutes of Health, UC4 DK104155 and the Juvenile Diabetes Research Foundation, JDRF 47-2013-520. Our research was also supported by the University of Turku Graduate School (UTUGS).

## Conflict of Interest

Author HH is employed by Fimlab Laboratories Ltd.

The remaining authors declare that the research was conducted in the absence of any commercial or financial relationships that could be construed as a potential conflict of interest.

## Publisher’s Note

All claims expressed in this article are solely those of the authors and do not necessarily represent those of their affiliated organizations, or those of the publisher, the editors and the reviewers. Any product that may be evaluated in this article, or claim that may be made by its manufacturer, is not guaranteed or endorsed by the publisher.
