# Supplementary material for: Pancreas Whole Tissue Transcriptomics Highlights the Role of the Exocrine Pancreas in Patients With Recently Diagnosed Type 1 Diabetes
Source: Front Endocrinol (Lausanne). 2022 Apr 13;13:861985. doi: 10.3389/fendo.2022.861985 (PMC9044038; doi:10.3389/fendo.2022.861985)
Supplement: Supplementary file 1 [file Presentation_1.pdf]

# *Supplementary Material*

## 1 RNA-seq analysis

### The whole pancreas transcriptomes 1 and 2

Preprocessing

The need for trimming of the raw RNA-seq reads was assessed with the high throughput quality control tool FastQC [1]. Low quality reads in both whole pancreas transcriptome datasets were trimmed using the TrimGalore! [2] software tool with the default settings: Quality Phred score cutoff 20, Illumina TruSeq, Sanger iPCR; auto-detected adapter sequences, Stringency 1 bp and 20 bp minimum required sequence length for both reads.

The trimmed RNA-seq reads were mapped to the University of California, Santa Cruz (UCSC) human reference genome Genome Reference Consortium Human Build 38 (GRCh38) [3] using the STAR aligner [4] version 2.5.2b. The featureCounts tool [5] included in the Subread [6] software package was applied to generate the read counts.

For further analysis, the lowly expressed genes were filtered using the uniquely mapped reads. For the differential expression analysis of the whole pancreas transcriptome 1, only genes with at least a count per million (cpm) expression over the threshold value in at least three samples (corresponding to the smallest experimental group size) were retained in the analysis. The threshold value used in the analysis was the cpm value corresponding to a read count value of 10 in the sample with the smallest library size [7]. For examining the differences in the gene expression between the Diabetes Virus Detection (DiViD) cases, a gene was required to have a cpm expression over the same threshold value in at least one sample to be retained in the analysis. Both whole pancreas transcriptomic datasets were processed similarly. The trimmed mean of M-values (TMM) normalization from the Bioconductor package edgeR [7, 8] was used to normalize the filtered gene counts. After normalization, the datasets were transformed to cpm, offsetted by 1 and $\log_2$ transformed.

## 2 RNA Microarray analysis

### The pancreatic islet transcriptome

Data preparation

The laser-capture microarray data was generated and normalized as described before by [9, 10].

Preprocessing

All probes not related to the experimental design were excluded from further analysis of the normalized annotated data using the annotation (HuGene-2_0-st-v1, release 36) downloaded from the Thermo Fisher (AffyMetrix) web site. Following, the data was $\log_2$ transformed and filtered for lowly expressed genes. As much as 50% of the probes in the microarray data can be expressing a background noise signal instead of a true signal [11]. Filtering of the noisy measurements prior to

differential expression (DE) analysis is recommended [11–13]. To retain a proper FDR control in the DE analysis, the filtering should be independent (i.e. blind) of the group labels of the samples [12, 13]. The pancreatic islet microarray data was thus filtered to retain only probes with expression larger than the median expression in the whole data in at least five samples (the size of the smallest experimental group) for further analysis. Similar to the filtering of RNASeq data, the group labels were not used for the low expression filtering satisfying the marginal independence criterion [12].

After low expression filtering, the microarray data was summarized to gene level by selecting a representative probe set for each gene in the filtered data. The selected probe set for each gene was the probe set with the largest mean intensity over all the samples.

## 3 Additional details regarding the data analysis of the preprocessed datasets

All data analyses of the preprocessed datasets were performed using the R statistical programming software environment version 3.6.0 [14].

Comparison of the DiViD cases

To compare the expression of genes between the DiViD cases, the gene expression in each dataset was z-score standardized. Furthermore, to allow for the comparison of expression patterns between cases over the examined different datasets, only the DiViD cases (not the varying non-diabetic organ donor controls) were used in the z-score standardization within each dataset. Thus, the z-score standardized expression $z_{ij}$ for gene *i* in sample *j* in any examined dataset was defined as:

$$z_{ij} = \frac{x_{ij} - \mu_{icases}}{\sigma_{icases}}$$

where $x_{ij}$ is the expression of gene *i* in sample *j*, $\mu_{icases}$ is the averaged expression of gene *i* over the DiViD cases and $\sigma_{icases}$ is the standard deviation of gene *i* over the DiViD cases.

Cell type proportion estimation

Following the recommendations in the CIBERSORT software, the quantile normalization option was disabled for the RNASeq dataset. Measurements from the acinar, alpha, beta, delta, and ductal pancreatic cell types of 20-40 year old individuals were utilized, excluding cells labeled as low quality.

Functional enrichment analysis

For Figure 3A, the expression of the differentially expressed genes in the whole pancreas transcriptome 1 and the pancreatic islet transcriptome involved in the terms GO:0006955 (immune response) and GO:0006954 (inflammatory response) were explored and compared.

To determine the subcellular localization of gene products in the terms GO:0006955 (immune response) and GO:0006954 (inflammatory response) for Figure 3B, Ingenuity Pathway Analysis (IPA) (QIAGEN Inc.) [15] was applied. The interactions, expression and subcellular localization among the immune and inflammatory response gene products for Figure 3B were visualized using Cytoscape version 3.7.2 [16].

# 4 Supplementary References


1. Andrews S, Krueger F, Segonds-Pichon A, Biggins L, Krueger C, Wingett S (2012) {FastQC}

2. Krueger F (2012) {Trim Galore!}

3. Schneider VA, Graves-Lindsay T, Howe K, et al (2017) Evaluation of GRCh38 and de novo haploid genome assemblies demonstrates the enduring quality of the reference assembly. Genome Res 27(5):849–864. https://doi.org/10.1101/gr.213611.116

4. Dobin A, Davis CA, Schlesinger F, et al (2013) STAR: ultrafast universal RNA-seq aligner. Bioinformatics 29(1):15–21. https://doi.org/10.1093/bioinformatics/bts635

5. Liao Y, Smyth GK, Shi W (2014) featureCounts: an efficient general purpose program for assigning sequence reads to genomic features. Bioinformatics 30(7):923–930. https://doi.org/10.1093/bioinformatics/btt656

6. Liao Y, Smyth GK, Shi W (2013) The Subread aligner: fast, accurate and scalable read mapping by seed-and-vote. Nucleic Acids Res 41(10):e108. https://doi.org/10.1093/nar/gkt214

7. Robinson MD, McCarthy DJ, Smyth GK (2010) edgeR: a Bioconductor package for differential expression analysis of digital gene expression data. Bioinformatics 26(1):139–140. https://doi.org/10.1093/bioinformatics/btp616

8. McCarthy DJ, Chen Y, Smyth GK (2012) Differential expression analysis of multifactor RNA-Seq experiments with respect to biological variation. Nucleic Acids Res 40(10):4288–4297. https://doi.org/10.1093/nar/gks042

9. Holm LJ, Krogvold L, Hasselby JP, et al (2018) Abnormal islet sphingolipid metabolism in type 1 diabetes. Diabetologia 61(7):1650–1661. https://doi.org/10.1007/s00125-018-4614-2

10. Richardson SJ, Rodriguez-Calvo T, Gerling IC, et al (2016) Islet cell hyperexpression of HLA class I antigens: a defining feature in type 1 diabetes. Diabetologia 59(11):2448–2458. https://doi.org/10.1007/s00125-016-4067-4

11. Hackstadt AJ, Hess AM (2009) Filtering for increased power for microarray data analysis. BMC Bioinformatics 10:11. https://doi.org/10.1186/1471-2105-10-11

12. Bourgon R, Gentleman R, Huber W (2010) Independent filtering increases detection power for high-throughput experiments. Proc Natl Acad Sci U S A 107(21):9546–9551. https://doi.org/10.1073/pnas.0914005107

13. Marczyk M, Jaksik R, Polanski A, Polanska J (2013) Adaptive filtering of microarray gene expression data based on Gaussian mixture decomposition. BMC Bioinformatics 14:101. https://doi.org/10.1186/1471-2105-14-101

14. R Core Team (2019) R: A Language and Environment for Statistical Computing

15. Kramer A, Green J, Pollard JJ, Tugendreich S (2014) Causal analysis approaches in Ingenuity Pathway Analysis. Bioinformatics 30(4):523–530.

https://doi.org/10.1093/bioinformatics/btt703

16. Shannon P, Markiel A, Ozier O, et al (2003) Cytoscape: a software environment for integrated models of biomolecular interaction networks. Genome Res 13(11):2498–2504. https://doi.org/10.1101/gr.1239303

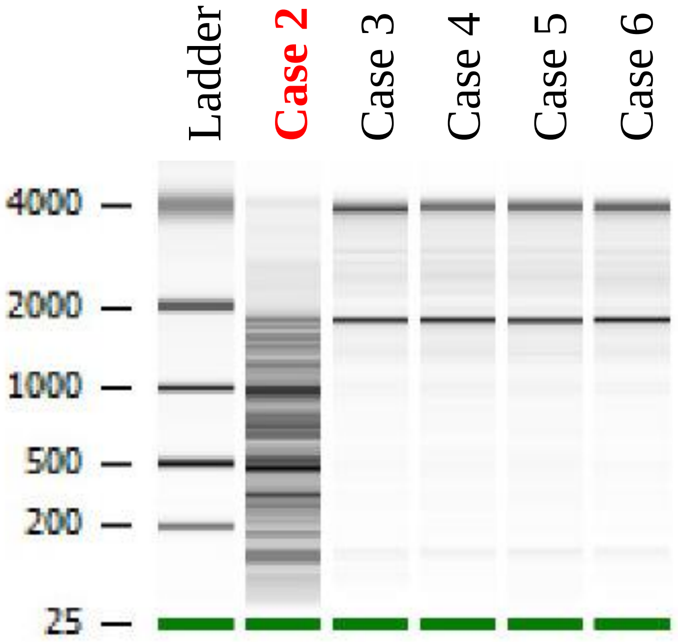


**Supplementary Figure 1.** RNA for the DiViD case 2 was degraded, and an adjusted protocol was used in the RNA-sequencing analysis of the sample.

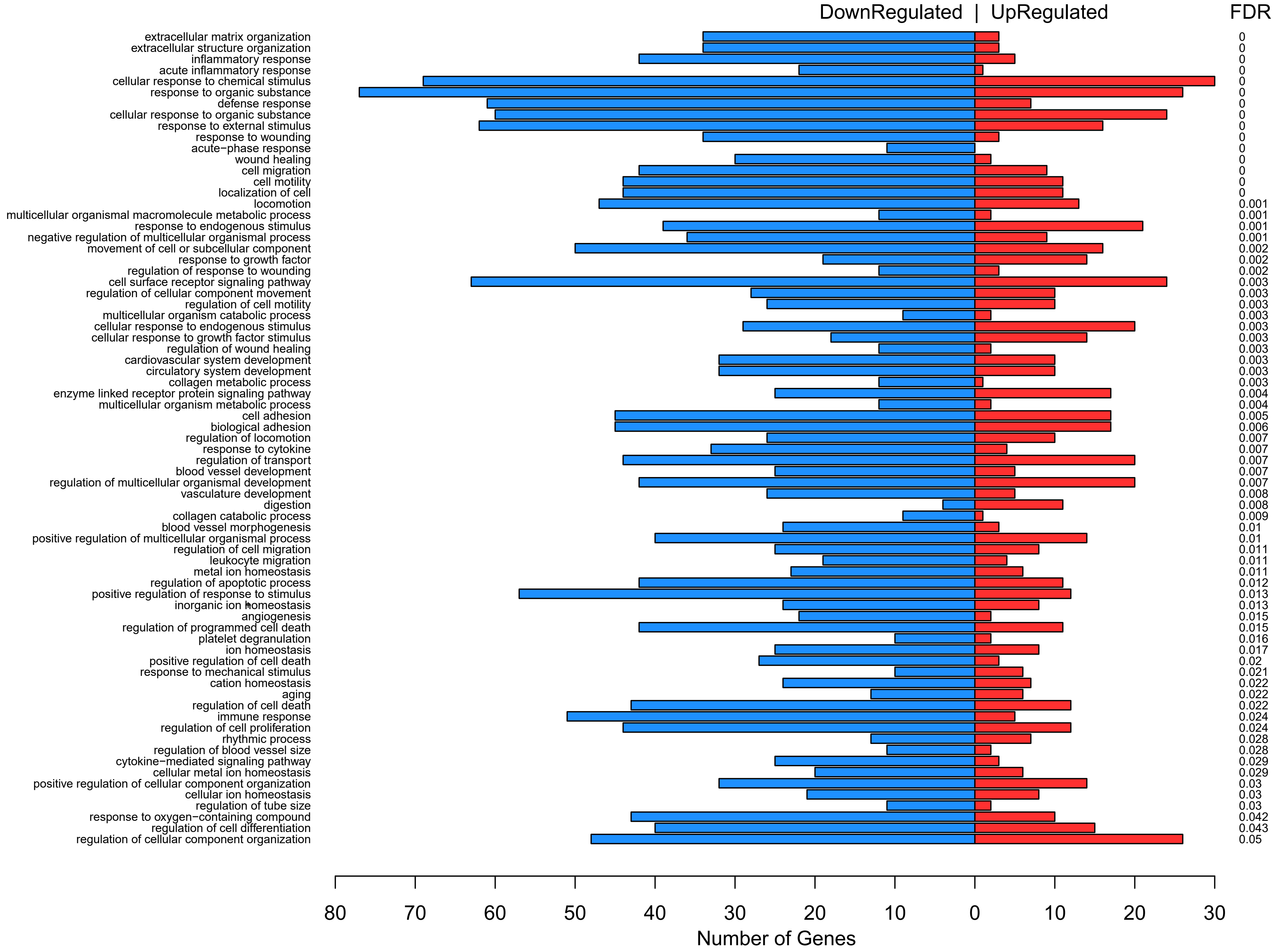


**Supplementary Figure 2.** The enriched GO FAT biological processes (BP) among the differentially expressed genes in whole pancreas transcriptome 1. The DE genes have been identified using the reproducibility optimized test statistic (ROTS) with a false discovery rate of ≤ 0.05 and |FC| ≥ 2 . Functional enrichment within the DE genes was examined using the Database for Annotation, Visualization and Integrated Discovery (DAVID).

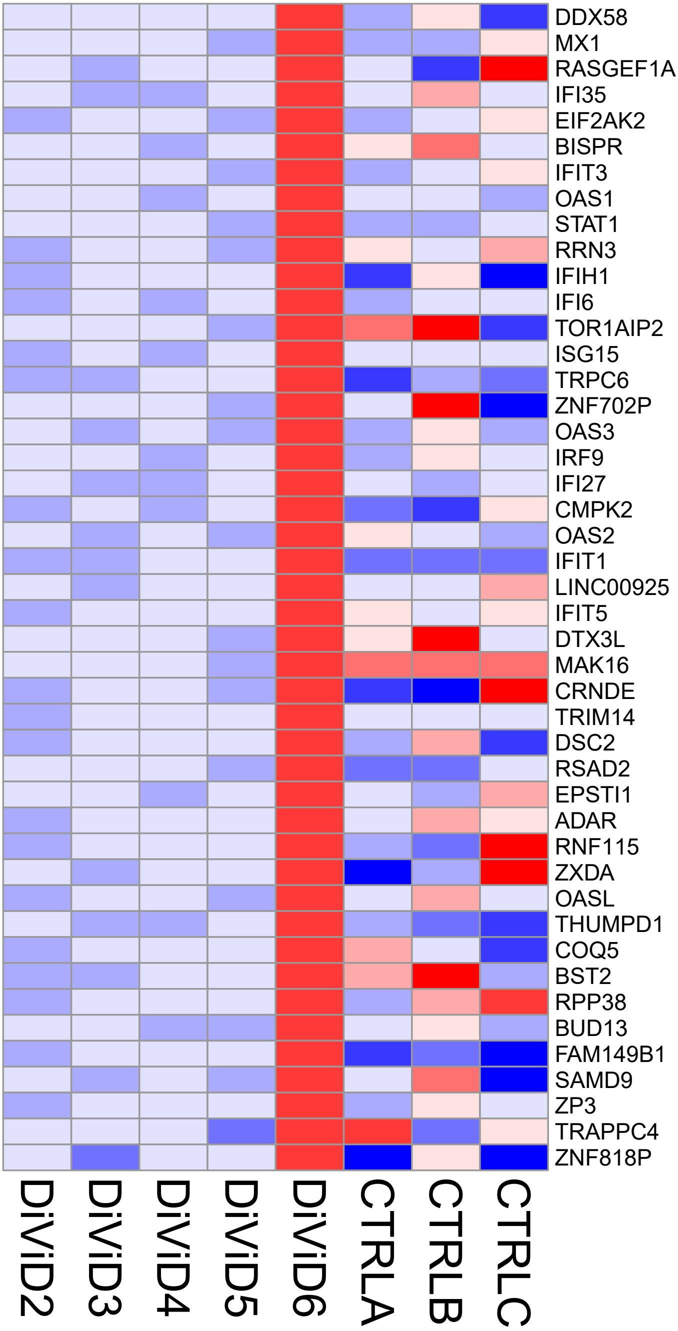


**Supplementary Figure 3**. Related to Figure 4A. A signature of 45 genes was discovered differentiating DiViD case 6 from the other DiViD patients and also the non-diabetic organ donor controls. The Z-score standardized expression of these genes is shown in the whole pancreas transcriptome 1.

**Supplementary Table 1.** The Commercial Human Pancreas total RNA preparates used as controls for RNA sequencing in the whole pancreas transcriptome 1.

| **Sample ID** | **Commercial pancreas tissue RNA preparate** | **Gender** | **Age (years)** | **Cause of death** |
|---|---|---|---|---|
| CTRL A | Ambion FirstChoice Human Pancreas Total RNA, #AM7954 | Male | 78 | Cardiac arrest |
| CTRL B | Agilent Technologies MVP Total RNA, Human Pancreas, #540023 | Female | 76 | NA |
| CTRL C | Clontech, Human Pancreas Total RNA #636577 | Male | 35 | Sudden death |

**Supplementary Table 2.** The differentially expressed (DE) genes between the DiViD cases and the controls in the whole pancreas transcriptome 1. The DE genes have been identified using the reproducibility optimized test statistic (ROTS) with a false discovery rate (FDR) of $\leq 0.05$ and $|FC| \geq 2$ . Additionally, the FDR between the DiViD cases and controls are shown for the same genes when the DE analysis is performed with the partly degraded DiViD case 2 excluded. CPM refers to Counts Per Million, logFC to logarithmic Fold Change (log2 transformed CPM data) and FDR to False Discovery Rate.

| Gene Symbol | CPM LogFC | FDR | FDR Case 2 Discarded |
|---|---|---|---|
| FOSB | 4.454 | 0.000 | 0.000 |
| GDF15 | -3.392 | 0.000 | 0.000 |
| CDKN1A | -2.877 | 0.000 | 0.000 |
| ARNTL | -2.252 | 0.000 | 0.000 |
| NNMT | -2.888 | 0.000 | 0.000 |
| EGR1 | 2.266 | 0.000 | 0.000 |
| REG3A | -5.680 | 0.000 | 0.000 |
| CLDN2 | -3.089 | 0.000 | 0.000 |
| CHAD | 1.958 | 0.000 | 0.000 |
| PTX3 | -2.108 | 0.000 | 0.000 |
| FN1 | -2.462 | 0.000 | 0.000 |
| TNC | -2.130 | 0.000 | 0.000 |
| PAPPA | -1.914 | 0.000 | 0.000 |
| ATP4A | 3.098 | 0.000 | 0.000 |
| C2CD4A | -2.274 | 0.000 | 0.000 |
| SERPINA3 | -3.857 | 0.000 | 0.000 |
| FREM1 | 1.983 | 0.000 | 0.000 |
| VCAN | -2.718 | 0.000 | 0.000 |
| F11 | 1.866 | 0.000 | 0.017 |
| SIGLEC11 | 3.255 | 0.000 | 0.000 |
| EPN3 | 2.265 | 0.000 | 0.000 |
| APCS | -1.583 | 0.000 | 0.021 |
| AGT | -2.190 | 0.000 | 0.000 |
| ABAT | 2.207 | 0.000 | 0.000 |
| CYP1B1 | -2.141 | 0.000 | 0.017 |
| GREM1 | -2.145 | 0.000 | 0.000 |
| INHBB | -1.628 | 0.000 | 0.000 |
| THBS1 | -2.461 | 0.000 | 0.000 |
| HLA-DRB1 | -1.448 | 0.000 | 0.021 |
| NGFR | -1.611 | 0.000 | 0.021 |

| | | | |
|---|---|---|---|
| NOS1 | 1.600 | 0.000 | 0.021 |
| RAB31 | -1.560 | 0.000 | 0.026 |
| DBP | 2.543 | 0.000 | 0.000 |
| ADCYAP1 | -1.613 | 0.000 | 0.021 |
| THEMIS2 | -1.538 | 0.000 | 0.026 |
| FOS | 1.910 | 0.000 | 0.021 |
| DDB2 | -1.190 | 0.000 | 0.028 |
| TGFB3 | -1.491 | 0.000 | 0.021 |
| TPM3 | 2.056 | 0.000 | 0.000 |
| AKR7A3 | -2.098 | 0.000 | 0.021 |
| DRAM1 | -1.383 | 0.000 | 0.028 |
| LAPTM5 | -1.887 | 0.000 | 0.021 |
| DES | -2.232 | 0.000 | 0.021 |
| PTP4A1 | -1.593 | 0.000 | 0.021 |
| GAREM | -1.536 | 0.000 | 0.021 |
| GCNT4 | 1.433 | 0.000 | 0.026 |
| TNFRSF12A | -1.762 | 0.000 | 0.026 |
| CYP3A4 | -2.501 | 0.000 | 0.021 |
| PER3 | 2.060 | 0.000 | 0.021 |
| CTRL | 2.670 | 0.000 | 0.000 |
| LINC00963 | -1.605 | 0.000 | 0.028 |
| TIMP1 | -2.175 | 0.000 | 0.021 |
| EEF1A2 | -1.590 | 0.000 | 0.028 |
| MYPN | 1.774 | 0.000 | 0.026 |
| SYCP2 | 2.410 | 0.000 | 0.017 |
| COL6A3 | -1.436 | 0.000 | 0.028 |
| RNASE1 | 3.293 | 0.000 | 0.000 |
| TMOD1 | -1.538 | 0.000 | 0.028 |
| PIEZO2 | -1.360 | 0.000 | 0.028 |
| LDHA | -1.786 | 0.000 | 0.028 |
| VDR | -1.244 | 0.000 | 0.033 |
| ALDOB | -3.421 | 0.000 | 0.017 |
| NMUR1 | 1.904 | 0.008 | 0.021 |
| ADD3 | 1.525 | 0.008 | 0.021 |
| AEN | -1.503 | 0.008 | 0.028 |
| B4GALT1 | -1.255 | 0.008 | 0.028 |
| EGF | 1.999 | 0.008 | 0.021 |
| LFNG | 1.547 | 0.008 | 0.028 |
| MSR1 | -1.700 | 0.008 | 0.028 |
| MMP2 | -1.581 | 0.008 | 0.028 |
| NPTX2 | -1.892 | 0.008 | 0.026 |
| CABP7 | -1.242 | 0.008 | 0.029 |
| CEMIP | -1.475 | 0.008 | 0.028 |
| KRT7 | -1.626 | 0.008 | 0.021 |
| SAT1 | -1.552 | 0.008 | 0.000 |
| MEP1A | 1.833 | 0.008 | 0.017 |
| IL10RA | -1.458 | 0.008 | 0.028 |

| | | | |
|---|---|---|---|
| CNN1 | -1.409 | 0.008 | 0.029 |
| C11orf96 | -2.640 | 0.008 | 0.021 |
| SMOC1 | -1.873 | 0.008 | 0.021 |
| TTYH1 | 1.320 | 0.008 | 0.029 |
| NPHS1 | 1.422 | 0.008 | 0.029 |
| ANKRD22 | -1.799 | 0.008 | 0.000 |
| ZNF737 | 1.187 | 0.008 | 0.035 |
| MUC5B | -2.572 | 0.008 | 0.026 |
| FAM159B | -1.022 | 0.008 | 0.035 |
| CD44 | -1.656 | 0.008 | 0.028 |
| PLCB1 | 1.068 | 0.008 | 0.035 |
| AQP12A | 1.730 | 0.008 | 0.028 |
| ADAMTS4 | -2.322 | 0.008 | 0.021 |
| LEFTY1 | 1.553 | 0.008 | 0.029 |
| FCGR3A | -1.937 | 0.008 | 0.028 |
| FAM107B | -1.011 | 0.008 | 0.036 |
| FGG | -2.195 | 0.008 | 0.026 |
| LGALS2 | 2.669 | 0.008 | 0.000 |
| C1S | -1.357 | 0.008 | 0.035 |
| FGA | -3.327 | 0.008 | 0.021 |
| VCAM1 | -1.424 | 0.008 | 0.035 |
| ACTA2 | -1.562 | 0.008 | 0.029 |
| C4BPA | -1.746 | 0.008 | 0.028 |
| FOSL2 | -1.449 | 0.008 | 0.028 |
| NWD1 | 1.098 | 0.008 | 0.036 |
| COL3A1 | -1.175 | 0.008 | 0.035 |
| HOGA1 | 1.351 | 0.008 | 0.035 |
| CRLF1 | -1.276 | 0.008 | 0.035 |
| LTBP4 | 1.303 | 0.008 | 0.035 |
| CRP | -2.644 | 0.008 | 0.026 |
| DEFB1 | -1.530 | 0.008 | 0.029 |
| PNLIPRP2 | 3.945 | 0.008 | 0.000 |
| RBBP8NL | 1.283 | 0.008 | 0.035 |
| LMO3 | 2.071 | 0.008 | 0.017 |
| REG1B | -4.268 | 0.008 | 0.021 |
| CP | -2.411 | 0.008 | 0.026 |
| ANGPTL4 | -2.155 | 0.008 | 0.000 |
| SLITRK1 | 1.246 | 0.008 | 0.035 |
| FHOD3 | 1.152 | 0.008 | 0.035 |
| C1R | -1.417 | 0.008 | 0.035 |
| CXCR4 | -1.573 | 0.008 | 0.028 |
| NCKAP1L | -1.256 | 0.008 | 0.035 |
| SGK1 | 1.717 | 0.008 | 0.029 |
| ITIH4 | -2.509 | 0.008 | 0.000 |
| TMEM2 | -1.104 | 0.008 | 0.035 |
| CITED2 | 1.578 | 0.013 | 0.021 |
| PDZK1 | 1.487 | 0.013 | 0.029 |

| | | | |
|---|---|---|---|
| THBS2 | -1.527 | 0.013 | 0.035 |
| SCN1B | -1.346 | 0.013 | 0.035 |
| ARNTL2 | -1.263 | 0.013 | 0.035 |
| NRG4 | 1.502 | 0.013 | 0.017 |
| ACY3 | 1.599 | 0.013 | 0.028 |
| EPHX1 | 1.653 | 0.013 | 0.000 |
| STEAP4 | -1.811 | 0.013 | 0.026 |
| LOC102723344 | 1.758 | 0.013 | 0.029 |
| CFB | -2.007 | 0.013 | 0.028 |
| ING2 | 1.104 | 0.013 | 0.035 |
| MAP1LC3B | -1.154 | 0.013 | 0.035 |
| CD163 | -2.259 | 0.013 | 0.021 |
| SLC2A2 | -1.321 | 0.013 | 0.035 |
| PDPN | -1.361 | 0.013 | 0.035 |
| C1orf162 | -1.307 | 0.013 | 0.035 |
| AMY2B | 2.817 | 0.013 | 0.000 |
| NUCB2 | 1.787 | 0.013 | 0.000 |
| SGSM1 | 1.176 | 0.013 | 0.036 |
| COL8A1 | -1.017 | 0.013 | 0.041 |
| PFKFB2 | -1.398 | 0.013 | 0.035 |
| SLC2A3 | -1.516 | 0.013 | 0.029 |
| TOX | -1.023 | 0.013 | 0.041 |
| AMHR2 | 1.530 | 0.013 | 0.021 |
| TMC5 | -1.721 | 0.013 | 0.029 |
| TNFRSF10B | -1.420 | 0.013 | 0.021 |
| DUSP4 | -1.980 | 0.013 | 0.000 |
| CELA2B | 3.651 | 0.013 | 0.000 |
| GCAT | 1.698 | 0.013 | 0.017 |
| GEM | -1.107 | 0.013 | 0.041 |
| MT1H | 1.583 | 0.013 | 0.026 |
| SLC4A7 | -1.121 | 0.017 | 0.028 |
| USP2 | 1.282 | 0.017 | 0.035 |
| MPP1 | 1.152 | 0.017 | 0.035 |
| ADAMTS16 | 1.492 | 0.017 | 0.035 |
| MCAM | -1.331 | 0.017 | 0.035 |
| DIAPH1 | -1.100 | 0.017 | 0.035 |
| BCAT2 | 1.265 | 0.017 | 0.028 |
| BDH1 | 1.180 | 0.017 | 0.035 |
| SDC1 | -1.120 | 0.017 | 0.041 |
| SLC11A1 | -1.727 | 0.017 | 0.028 |
| ME1 | -1.274 | 0.017 | 0.035 |
| HLA-DQB1 | -1.824 | 0.017 | 0.017 |
| MT1G | 1.846 | 0.017 | 0.026 |
| BTG3 | -1.108 | 0.017 | 0.041 |
| DPP10 | 1.492 | 0.017 | 0.035 |
| PM20D1 | 1.993 | 0.017 | 0.033 |
| ERP27 | 2.218 | 0.017 | 0.000 |

| | | | |
|---|---|---|---|
| LAPTM4B | -1.061 | 0.017 | 0.037 |
| SMURF1 | -1.085 | 0.017 | 0.035 |
| EGR3 | 1.229 | 0.017 | 0.041 |
| CHST11 | -1.502 | 0.017 | 0.035 |
| MYH11 | -1.790 | 0.017 | 0.028 |
| G6PC2 | -2.219 | 0.017 | 0.021 |
| MOXD1 | -1.053 | 0.018 | 0.046 |
| FLJ38122 | 1.467 | 0.018 | 0.035 |
| PHLDA3 | -1.199 | 0.018 | 0.041 |
| SYCN | 2.813 | 0.018 | 0.017 |
| GABARAPL1 | -1.082 | 0.018 | 0.041 |
| ACTG2 | -1.287 | 0.018 | 0.035 |
| CTHRC1 | -1.186 | 0.018 | 0.037 |
| SHC3 | -1.538 | 0.018 | 0.035 |
| CES3 | 1.191 | 0.018 | 0.035 |
| PACSIN1 | 1.635 | 0.018 | 0.033 |
| COL4A2 | -1.111 | 0.018 | 0.028 |
| SYT13 | -1.250 | 0.018 | 0.035 |
| IRF8 | -1.296 | 0.018 | 0.041 |
| EGLN3 | -1.222 | 0.018 | 0.041 |
| CNIH3 | 1.423 | 0.018 | 0.021 |
| F3 | -1.594 | 0.018 | 0.035 |
| FPR1 | -1.500 | 0.018 | 0.035 |
| IZUMO4 | 1.182 | 0.018 | 0.044 |
| F2R | -1.049 | 0.018 | 0.046 |
| SERPINI2 | 1.750 | 0.018 | 0.000 |
| GALT | 1.034 | 0.018 | 0.050 |
| HLA-DQA1 | -1.004 | 0.018 | 0.037 |
| SYNPO2 | -1.406 | 0.018 | 0.035 |
| MMP1 | -1.164 | 0.018 | 0.041 |
| PDGFRA | -1.070 | 0.018 | 0.046 |
| CLPS | 2.532 | 0.018 | 0.000 |
| YIPF4 | 1.117 | 0.018 | 0.028 |
| ASAH2 | 1.270 | 0.018 | 0.035 |
| LOC101927188 | 1.119 | 0.018 | 0.029 |
| PLEKHG1 | -1.138 | 0.018 | 0.028 |
| TMED11P | 1.984 | 0.021 | 0.021 |
| GUCA1C | 1.726 | 0.021 | 0.017 |
| SLC6A17 | -1.155 | 0.021 | 0.041 |
| ELOVL5 | -1.098 | 0.021 | 0.037 |
| ACKR1 | -1.110 | 0.021 | 0.041 |
| LMOD1 | -1.199 | 0.021 | 0.035 |
| OLFML2B | -1.000 | 0.021 | 0.051 |
| PAPLN | -1.170 | 0.021 | 0.028 |
| PLCH2 | -1.564 | 0.021 | 0.029 |
| APOC1 | -1.313 | 0.021 | 0.041 |
| ASCC1 | -1.087 | 0.021 | 0.046 |

| | | | |
|---|---|---|---|
| USP51 | 1.072 | 0.021 | 0.051 |
| TACSTD2 | -1.281 | 0.021 | 0.041 |
| C1QC | -1.495 | 0.021 | 0.037 |
| NFIL3 | -1.604 | 0.021 | 0.035 |
| CTRB2 | 1.686 | 0.021 | 0.028 |
| GPX3 | -1.128 | 0.021 | 0.049 |
| FSTL3 | -1.620 | 0.021 | 0.028 |
| BBC3 | -1.045 | 0.024 | 0.051 |
| TET1 | 1.001 | 0.024 | 0.055 |
| NDST1 | 1.011 | 0.024 | 0.035 |
| EMILIN2 | -1.133 | 0.024 | 0.046 |
| KIAA0040 | -1.398 | 0.024 | 0.041 |
| OSMR | -1.614 | 0.024 | 0.028 |
| DOC2B | -1.428 | 0.024 | 0.035 |
| LINC01251 | 1.545 | 0.024 | 0.041 |
| MVP | -1.190 | 0.024 | 0.043 |
| MT1F | 1.347 | 0.024 | 0.028 |
| B4GALT5 | -1.080 | 0.024 | 0.050 |
| WBSCR17 | 1.404 | 0.027 | 0.028 |
| ANGPTL1 | 1.442 | 0.027 | 0.036 |
| BACH2 | -1.124 | 0.027 | 0.029 |
| NUPR1 | 1.629 | 0.027 | 0.035 |
| VEPH1 | 1.017 | 0.027 | 0.059 |
| ICAM1 | -1.664 | 0.027 | 0.035 |
| ABHD5 | -1.120 | 0.029 | 0.046 |
| C5AR1 | -1.419 | 0.029 | 0.037 |
| PDIA2 | 1.718 | 0.029 | 0.028 |
| CREB3L1 | -1.263 | 0.029 | 0.046 |
| PIGR | -1.526 | 0.029 | 0.041 |
| KIF5C | -1.151 | 0.029 | 0.035 |
| TNFRSF1B | -1.188 | 0.029 | 0.043 |
| DNASE1 | 2.358 | 0.029 | 0.028 |
| GALNT11 | 1.328 | 0.029 | 0.028 |
| LCP1 | -1.403 | 0.029 | 0.041 |
| GPHA2 | 2.666 | 0.029 | 0.000 |
| F13A1 | -1.858 | 0.029 | 0.035 |
| CSGALNACT1 | -1.131 | 0.029 | 0.041 |
| KDM8 | 1.083 | 0.029 | 0.051 |
| SLC3A2 | -1.301 | 0.029 | 0.044 |
| SLC16A3 | -1.391 | 0.029 | 0.041 |
| SLC16A10 | 1.365 | 0.029 | 0.028 |
| TEX11 | 2.554 | 0.029 | 0.000 |
| LCN2 | -1.826 | 0.029 | 0.036 |
| MMP7 | -1.849 | 0.029 | 0.036 |
| HRASLS5 | 1.733 | 0.029 | 0.000 |
| CCDC186 | 1.093 | 0.029 | 0.051 |
| AQP9 | -1.250 | 0.029 | 0.046 |

| | | | |
|---|---|---|---|
| ADAMTS2 | -1.004 | 0.029 | 0.051 |
| INSIG1 | 1.582 | 0.029 | 0.000 |
| REG1P | -4.139 | 0.029 | 0.028 |
| ACE | 1.251 | 0.029 | 0.041 |
| SCNN1G | -1.096 | 0.029 | 0.041 |
| ZMAT3 | -1.112 | 0.029 | 0.050 |
| SULF2 | -1.383 | 0.029 | 0.041 |
| AQP11 | 1.646 | 0.029 | 0.017 |
| SLC7A5 | -1.577 | 0.029 | 0.035 |
| FCGR2A | -1.510 | 0.029 | 0.041 |
| IGFBP4 | -1.537 | 0.029 | 0.033 |
| COL6A6 | 1.134 | 0.029 | 0.051 |
| ID3 | -1.292 | 0.031 | 0.037 |
| PTGER4 | 1.832 | 0.031 | 0.035 |
| COL4A1 | -1.260 | 0.031 | 0.029 |
| RRBP1 | 1.004 | 0.031 | 0.041 |
| IL15RA | -1.095 | 0.031 | 0.041 |
| HMOX1 | -1.059 | 0.031 | 0.053 |
| RHBG | 1.097 | 0.031 | 0.050 |
| PDZD8 | -1.048 | 0.031 | 0.051 |
| ZNF302 | 1.126 | 0.031 | 0.055 |
| CRISPLD2 | -1.007 | 0.031 | 0.035 |
| SLC8A2 | 1.348 | 0.031 | 0.021 |
| APOE | -1.588 | 0.031 | 0.037 |
| TYROBP | -1.072 | 0.031 | 0.057 |
| IGFBP2 | 2.349 | 0.033 | 0.021 |
| SERPINA1 | -1.606 | 0.033 | 0.035 |
| STAT3 | -1.083 | 0.033 | 0.055 |
| IRF1 | -1.331 | 0.033 | 0.044 |
| S100A9 | -1.716 | 0.033 | 0.041 |
| CRY1 | -1.044 | 0.033 | 0.055 |
| LRRTM3 | -1.192 | 0.038 | 0.041 |
| RBPJL | 1.800 | 0.038 | 0.037 |
| CENPC | 1.157 | 0.038 | 0.055 |
| NPY1R | 1.536 | 0.038 | 0.046 |
| CELA3A | 1.776 | 0.038 | 0.026 |
| HPX | -1.365 | 0.038 | 0.041 |
| DDIT4 | -1.548 | 0.038 | 0.035 |
| S1PR5 | 1.120 | 0.038 | 0.035 |
| EFEMP1 | -1.034 | 0.038 | 0.065 |
| MAFF | -1.013 | 0.038 | 0.046 |
| AGA | 1.463 | 0.038 | 0.028 |
| RARRES2 | 1.728 | 0.038 | 0.028 |
| CXCL2 | -2.230 | 0.038 | 0.035 |
| CNKSR2 | 1.005 | 0.038 | 0.069 |
| GBP2 | -1.072 | 0.038 | 0.053 |
| MYCL | 2.103 | 0.038 | 0.035 |

| | | | |
|---|---|---|---|
| AMY2A | 2.548 | 0.038 | 0.026 |
| POSTN | -1.242 | 0.038 | 0.053 |
| WNK2 | 1.459 | 0.038 | 0.046 |
| DGCR5 | 1.166 | 0.038 | 0.051 |
| EPHA8 | 1.419 | 0.038 | 0.021 |
| RN7SL1 | 1.080 | 0.038 | 0.069 |
| REG3G | -3.186 | 0.039 | 0.035 |
| SLC7A1 | -1.017 | 0.039 | 0.068 |
| DMD | 1.002 | 0.039 | 0.046 |
| MAP1B | -1.212 | 0.039 | 0.035 |
| SPON1 | -1.097 | 0.039 | 0.064 |
| PALD1 | -1.029 | 0.042 | 0.035 |
| MSC-AS1 | -1.059 | 0.042 | 0.068 |
| RGMA | 1.059 | 0.042 | 0.069 |
| RNA45S5 | 1.772 | 0.042 | 0.041 |
| PPP1R10 | 1.114 | 0.042 | 0.065 |
| KLK11 | 1.881 | 0.042 | 0.026 |
| SPSB4 | 1.314 | 0.042 | 0.029 |
| CTRC | 2.261 | 0.042 | 0.028 |
| SOD2 | -1.856 | 0.042 | 0.043 |
| PEX5L | 1.230 | 0.042 | 0.063 |
| FGFRL1 | 1.095 | 0.042 | 0.062 |
| TGM2 | -2.064 | 0.042 | 0.041 |
| OLFML2A | 1.217 | 0.042 | 0.037 |
| PSMD5-AS1 | 1.180 | 0.042 | 0.063 |
| C3 | -1.970 | 0.042 | 0.036 |
| CD68 | -1.172 | 0.042 | 0.063 |
| KSR1 | -1.281 | 0.042 | 0.028 |
| C1QB | -1.692 | 0.042 | 0.047 |
| LENG9 | 1.024 | 0.046 | 0.035 |
| SIK1 | -1.388 | 0.046 | 0.021 |
| TEF | 1.183 | 0.046 | 0.041 |
| DDX21 | -1.036 | 0.046 | 0.069 |
| AQP12B | 2.108 | 0.046 | 0.036 |
| SAA1 | -1.177 | 0.048 | 0.063 |
| TFF2 | -1.133 | 0.048 | 0.062 |
| OBSCN | 1.216 | 0.048 | 0.065 |
| PNPLA7 | 1.457 | 0.048 | 0.051 |
| APLP1 | 1.155 | 0.048 | 0.035 |
| ANKRD62 | 1.194 | 0.048 | 0.050 |
| RGS1 | -1.639 | 0.048 | 0.036 |
| GCNT3 | -1.458 | 0.049 | 0.047 |
| FAM105A | -1.083 | 0.049 | 0.035 |
| ENPP1 | 1.532 | 0.049 | 0.029 |
| FAM46C | 1.328 | 0.049 | 0.028 |
| PALLD | -1.283 | 0.049 | 0.035 |
| LAMB1 | -1.117 | 0.049 | 0.041 |

| | | | |
|---|---|---|---|
| C1orf127 | -1.116 | 0.049 | 0.041 |
| PBLD | 1.159 | 0.049 | 0.041 |
| TGFBI | -1.037 | 0.049 | 0.069 |
| DMBT1 | -1.277 | 0.049 | 0.059 |
| RAB3B | -1.412 | 0.049 | 0.035 |
| CDHR5 | 1.089 | 0.049 | 0.043 |

**Supplementary Table 3 related to Figure 3A.** Immune and inflammatory responses in the whole pancreas transcriptome 1 and the pancreatic islet transcriptome. The up-regulated and down-regulated immune and inflammatory response genes between the DiViD patients and the controls in each tissue type and those commonly regulated. The differentially expressed (DE) genes were identified using the reproducibility optimized test statistic (ROTS) [33] with a false discovery rate of ≤ 0.05 and |FC| ≥ 2. Genes in the biological processes GO:0006955 (immune response) and GO:0006954 (inflammatory response) were examined.

| UpRegulated genes whole tissue transcriptome 1 | DownRegulated genes whole tissue transcriptome 1 | UpRegulated genes islet transcriptome | DownRegulated genes islet transcriptome | Commonly UpRegulated | Commonly DownRegulated |
|---|---|---|---|---|---|
| FOS | THEMIS2 | CXCL9 | PSMD7 | EGR1 | APCS |
| EGR1 | APCS | STAT1 | SERPINA3 | | SERPINA3 |
| LFNG | AGT | CXCL10 | PSMB6 | | ADCYAP1 |
| NDST1 | TGFB3 | CCL5 | PRELID1 | | DEFB1 |
| NUPR1 | SERPINA3 | B2M | C6 | | CXCL2 |
| PTGER4 | THBS1 | CD74 | PRDX1 | | REG3G |
| RARRES2 | NGFR | DDX58 | AMBP | | |
| ENPP1 | ADCYAP1 | HLA-DQA2 | IGLL1 | | |
| | REG3A | RSAD2 | SHC1 | | |
| | FN1 | HLA-DQA1 | HIST1H2BD | | |
| | PTX3 | DDX60 | PSMD14 | | |
| | HLA-DRB1 | HLA-DRA | FFAR3 | | |
| | VCAM1 | HLA-DPA1 | SLPI | | |
| | CRP | MX1 | CD24 | | |
| | FCGR3A | HLA-E | BCL6 | | |
| | C4BPA | SLAMF7 | DEFB1 | | |
| | CD44 | PTPRC | SERPINF1 | | |
| | C1S | FYB | REG3G | | |
| | C1R | IFIT3 | ADCYAP1 | | |
| | NCKAP1L | GZMA | CXCL17 | | |
| | MMP2 | XAF1 | APCS | | |
| | CXCR4 | TRIM22 | SPP1 | | |
| | COL3A1 | IGKV1-39 | CXCL2 | | |
| | ITIH4 | GBP1 | NPY | | |
| | FGA | IGHG4 | | | |
| | DEFB1 | ISG15 | | | |
| | B4GALT1 | IL2RG | | | |
| | CD163 | TAP1 | | | |
| | CFB | IGSF6 | | | |
| | TNFRSF10B | HLA-A | | | |
| | GEM | IGKC | | | |
| | SDC1 | IGKV1-16 | | | |

| | | | | | |
|---|---|---|---|---|---|
| | SLC11A1 | CD3D | | | |
| | HLA-DQB1 | CCL4L1 | | | |
| | F3 | LCP2 | | | |
| | IRF8 | HLA-B | | | |
| | FPR1 | IGHG1 | | | |
| | F2R | IGKV3-15 | | | |
| | HLA-DQA1 | CCL4 | | | |
| | C1QC | IGHM | | | |
| | ACKR1 | IGLV1-44 | | | |
| | NFIL3 | IFI6 | | | |
| | OSMR | CXCR4 | | | |
| | ICAM1 | RGS1 | | | |
| | TNFRSF1B | EGR1 | | | |
| | PIGR | CTSS | | | |
| | LCP1 | IGLC7 | | | |
| | C5AR1 | LYZ | | | |
| | FCGR2A | | | | |
| | AQP9 | | | | |
| | IGFBP4 | | | | |
| | LCN2 | | | | |
| | TYROBP | | | | |
| | APOE | | | | |
| | HMOX1 | | | | |
| | S100A9 | | | | |
| | SERPINA1 | | | | |
| | STAT3 | | | | |
| | IRF1 | | | | |
| | HPX | | | | |
| | GBP2 | | | | |
| | CXCL2 | | | | |
| | REG3G | | | | |
| | C1QB | | | | |
| | C3 | | | | |
| | TGM2 | | | | |
| | SAA1 | | | | |
| | RGS1 | | | | |
| | GCNT3 | | | | |
| | DMBT1 | | | | |

**Supplementary Data 1.** The utilized signature matrix for the estimation of the cell type proportions with the online deconvolution tool CIBERSORT in the whole pancreas transcriptome 1. The signature matrix was constructed from a pancreatic single-cell data under accession ID E-MTAB-5061 in ArrayExpress.

| Genes | acinar | alpha | beta | delta | ductal |
|---|---|---|---|---|---|
| CTRB2 | 7554.83929 | 0.33070866 | 0.21276596 | 0.375 | 11.0785714 |
| PRSS3P2 | 2699.98214 | 0.18110236 | 0.09219858 | 0.42857143 | 20.5071429 |
| CTRB1 | 3102.60714 | 0.17519685 | 0.12765957 | 0.23214286 | 4.21428571 |
| CPA1 | 4235.66071 | 1.33464567 | 3.39716312 | 1.10714286 | 10.5285714 |
| RNASE1 | 1565.55357 | 0.13385827 | 0.15602837 | 0.10714286 | 90.6142857 |
| CPA2 | 2222.30357 | 0.67519685 | 0.17021277 | 0.21428571 | 1.27857143 |
| CPB1 | 2891.08929 | 0.75393701 | 0.13475177 | 161.625 | 2.77142857 |
| DUOXA2 | 3259.80357 | 0.52559055 | 0.27659574 | 0.33928571 | 170.285714 |
| CXCL17 | 659.714286 | 0.11614173 | 0.04255319 | 0.07142857 | 9.83571429 |
| ALDOB | 1195.60714 | 0.82283465 | 1.34042553 | 0.05357143 | 10.5714286 |
| REG1B | 18938.6786 | 0.9015748 | 0.53191489 | 0.98214286 | 9.60714286 |
| PNLIP | 2741.17857 | 0.18700787 | 3.73049645 | 0.125 | 1.83571429 |
| REG1A | 79729.2321 | 4.35433071 | 8.34042553 | 6.16071429 | 608.478571 |
| REG3A | 25584.6786 | 1.47637795 | 1.04255319 | 1.48214286 | 6.34285714 |
| PRSS1 | 22717.6786 | 1.8011811 | 3.46099291 | 1.30357143 | 92.9642857 |
| MGST1 | 717.5 | 20.3444882 | 0.08510638 | 0.07142857 | 9.01428571 |
| SPINK1 | 10716.4286 | 65.7086614 | 0.9929078 | 1.39285714 | 102.35 |
| CTRC | 1155.83929 | 0.1003937 | 0.06382979 | 0.03571429 | 1.34285714 |
| PRSS3 | 4939.60714 | 25.0295276 | 0.26241135 | 2.25 | 36.9071429 |
| RARRES2 | 814.785714 | 0.0492126 | 0.09219858 | 0.05357143 | 20.9428571 |
| CELA3A | 2363.5 | 0.10629921 | 0.09219858 | 0.10714286 | 4.53571429 |
| OLFM4 | 7949.26786 | 0.72440945 | 1.23404255 | 1.21428571 | 895.121429 |
| GSTA2 | 596.714286 | 0.40944882 | 0.05673759 | 0.05357143 | 8.1 |
| ANPEP | 1501.14286 | 9.42125984 | 0.14893617 | 0.07142857 | 99.0714286 |
| PNLIPRP2 | 1183.08929 | 0.06692913 | 0.11347518 | 0.21428571 | 0.71428571 |
| GSTA1 | 772.696429 | 28.6161417 | 0.12765957 | 0.14285714 | 52.1285714 |
| LGALS2 | 714.625 | 0.06102362 | 0.07092199 | 0.01785714 | 15.1142857 |
| LDHB | 397.339286 | 7.73228346 | 46.8723404 | 0.78571429 | 5.25714286 |
| PLA2G1B | 645.803571 | 1.08661417 | 0.06382979 | 0.80357143 | 2.36428571 |
| UGDH | 726.678571 | 36.2047244 | 82.8085106 | 55.1428571 | 58.5285714 |
| AKR1C3 | 1057.03571 | 2.89173228 | 1.18439716 | 0.08928571 | 157.7 |
| DUOX2 | 1605.125 | 0.81889764 | 0.14184397 | 0.07142857 | 253.192857 |
| IL32 | 1463.32143 | 0.66338583 | 0.15602837 | 0.10714286 | 296.078571 |
| LYZ | 1020.78571 | 1.29724409 | 0.5248227 | 0.80357143 | 134.857143 |
| DPEP1 | 245.678571 | 3.96062992 | 0.02836879 | 0.10714286 | 0.07142857 |
| MUC1 | 1002.48214 | 11.3031496 | 0.38297872 | 3.25 | 210.592857 |
| C15orf48 | 1027.25 | 76.3425197 | 19.6099291 | 58.1428571 | 79.4642857 |
| GDF15 | 1357.85714 | 39.8779528 | 60.177305 | 96.4464286 | 559.235714 |
| SOD2 | 1421.78571 | 30.8543307 | 83.7446809 | 6.85714286 | 428.514286 |
| UBD | 655.428571 | 5.33267717 | 0.16312057 | 0.17857143 | 242.278571 |
| BCAT1 | 197.839286 | 0.23031496 | 0 | 0 | 0.08571429 |

| | | | | | |
|---|---|---|---|---|---|
| MT1G | 1455.26786 | 2.21259843 | 0.24113475 | 1.26785714 | 68.4571429 |
| DUSP4 | 224.982143 | 11.0413386 | 2.73758865 | 1.07142857 | 125.207143 |
| B3GNT7 | 263.375 | 2.01181102 | 1.5248227 | 1.83928571 | 33.6857143 |
| ANGPTL4 | 965.589286 | 17.2952756 | 1.02836879 | 0.16071429 | 139.992857 |
| CELA2A | 285.625 | 0.03149606 | 0.02836879 | 0.01785714 | 0.01428571 |
| PGM1 | 278.160714 | 44.8838583 | 50.6099291 | 23.6607143 | 48.2785714 |
| VIM | 325.125 | 80.2637795 | 0.44680851 | 39.625 | 111.428571 |
| TMC5 | 260.857143 | 1 | 0.19148936 | 0.33928571 | 110.8 |
| TPST2 | 162.928571 | 1.39370079 | 2.25531915 | 1.66071429 | 9.22142857 |
| GCG | 1488.57143 | 102567.211 | 189.255319 | 367.303571 | 2065.78571 |
| F10 | 3.33928571 | 208.919291 | 6.09219858 | 26.3214286 | 2.67142857 |
| SMIM24 | 37.125 | 201.700787 | 14.4680851 | 8.51785714 | 18.4642857 |
| GC | 62 | 984.385827 | 3.60992908 | 2.82142857 | 143.642857 |
| RGS4 | 20.9107143 | 256.76378 | 21.1276596 | 11.2142857 | 5.52857143 |
| TTR | 756.053571 | 36594.6693 | 2702.39007 | 1502.23214 | 538.057143 |
| CRYBA2 | 37.5714286 | 1155.5 | 80.9078014 | 34.7142857 | 12.65 |
| TM4SF4 | 60.6964286 | 3034.28346 | 59.7659574 | 329.196429 | 343.307143 |
| LOXL4 | 50.875 | 660.511811 | 0.4751773 | 0.82142857 | 30 |
| FAP | 4.76785714 | 139.564961 | 0.15602837 | 0.125 | 1.37142857 |
| VGF | 30.5535714 | 371.232283 | 16.0425532 | 7.42857143 | 6.29285714 |
| SERPINE2 | 10.3928571 | 127.761811 | 6.19858156 | 16.75 | 7.52857143 |
| CD99L2 | 18.2678571 | 112.338583 | 23.2836879 | 25.5178571 | 11.1357143 |
| PCSK2 | 31.3571429 | 1693.03937 | 225.120567 | 371.964286 | 26.9714286 |
| TMEM176B | 212.464286 | 725.240157 | 31.3687943 | 171.875 | 112.228571 |
| FXYD5 | 46.0357143 | 261.48622 | 2.53900709 | 6 | 49.7 |
| GLS | 41.8035714 | 192.409449 | 17.2553191 | 11.2678571 | 31.8285714 |
| SCG2 | 40.7678571 | 1168.29921 | 404.262411 | 337.017857 | 18.7857143 |
| TMEM176A | 115.392857 | 372.850394 | 13.5815603 | 124.785714 | 50.5642857 |
| SLC22A17 | 17.4642857 | 330.456693 | 48.0283688 | 100.732143 | 8.68571429 |
| PDK3 | 23.0714286 | 104.356299 | 45.6241135 | 9.14285714 | 17.05 |
| PLCE1 | 13.5357143 | 174.464567 | 0.58865248 | 0.25 | 5.49285714 |
| IRX2 | 0.96428571 | 69.8562992 | 0.07801418 | 0.07142857 | 2.06428571 |
| SLC38A4 | 2.23214286 | 134.17126 | 23.4255319 | 29.9285714 | 6.65714286 |
| CHGA | 56.8392857 | 2045.54724 | 701.134752 | 780.642857 | 52.4071429 |
| PALLD | 49.5 | 127.681102 | 19.787234 | 49.0178571 | 47.5357143 |
| ARX | 1.05357143 | 64.0590551 | 0.04255319 | 0.07142857 | 2.50714286 |
| PLK2 | 61.9285714 | 225.464567 | 77.2836879 | 70.0178571 | 78.1857143 |
| FEV | 2.55357143 | 77.4625984 | 0.56737589 | 8.83928571 | 2.42857143 |
| ALDH1A1 | 591.696429 | 1397.44882 | 483.049645 | 213.160714 | 246.292857 |
| CLU | 1379.73214 | 3898.45669 | 1077.77305 | 1262.26786 | 1290.35 |
| C10orf10 | 184.321429 | 1324.90157 | 162.546099 | 85.1071429 | 154.6 |
| FXYD3 | 94.875 | 183.704724 | 6.80141844 | 21.5 | 6.82857143 |
| SLC7A2 | 92.8035714 | 220.297244 | 49.2907801 | 36.8035714 | 4.71428571 |
| GRIA3 | 1.71428571 | 62.1181102 | 16.6241135 | 8.42857143 | 0.4 |
| GPX3 | 123.535714 | 1350.25591 | 223.546099 | 482.464286 | 45.4 |
| CHGB | 234.767857 | 9449.49606 | 2296.32624 | 4514.03571 | 76.8928571 |
| KCTD12 | 1.92857143 | 78.484252 | 0.41134752 | 20.6607143 | 7.6 |

| | | | | | |
|---|---|---|---|---|---|
| SMOC1 | 25.4285714 | 79.1102362 | 1.43262411 | 18.375 | 0.75 |
| CNTN1 | 1.57142857 | 123.222441 | 75.7234043 | 44.875 | 3.98571429 |
| CPLX2 | 1.23214286 | 92.4724409 | 32.212766 | 40.5892857 | 0.52857143 |
| PEMT | 47.4464286 | 625.824803 | 314.496454 | 96.2142857 | 41.1142857 |
| MOB1B | 6.03571429 | 77.1870079 | 33.751773 | 21.4642857 | 12.2357143 |
| SSX2IP | 6.33928571 | 70.8799213 | 20.9716312 | 19 | 7.50714286 |
| KIAA1324 | 133.321429 | 561.665354 | 299.985816 | 237.178571 | 8.53571429 |
| PDK4 | 75.5357143 | 366.21063 | 23.0780142 | 34.8571429 | 29.6642857 |
| PCDH17 | 1.71428571 | 56.1771654 | 18.5602837 | 17.2857143 | 6.59285714 |
| PTPRN2 | 35.5892857 | 244.470472 | 83.7234043 | 137.214286 | 35.0071429 |
| NENF | 42.4464286 | 155.757874 | 52.0212766 | 77.25 | 72.7142857 |
| SLC29A4 | 11.5357143 | 107.777559 | 34.7163121 | 46.4107143 | 10.8285714 |
| INS | 23.7142857 | 72.9507874 | 66312.9929 | 659.875 | 11.9 |
| IAPP | 3.08928571 | 3.98818898 | 4956.88652 | 12.1428571 | 1.09285714 |
| G6PC2 | 3.58928571 | 421.379921 | 1733.75177 | 11.9107143 | 0.80714286 |
| PFKFB2 | 24.4107143 | 16.8523622 | 352.631206 | 61.3392857 | 25.9214286 |
| ABCC8 | 12.5714286 | 317.690945 | 905.092199 | 440.053571 | 3.52857143 |
| NPTX2 | 19.9107143 | 11.3917323 | 302.765957 | 92.3392857 | 10.2285714 |
| ERO1LB | 61 | 244.393701 | 754.985816 | 252.375 | 7.98571429 |
| EIF4A2 | 191.517857 | 180.253937 | 441.22695 | 227.678571 | 159.028571 |
| RASD1 | 5.76785714 | 99 | 257.524823 | 94.4285714 | 45.1571429 |
| FAM159B | 0.10714286 | 28.8917323 | 200.347518 | 29.125 | 0.35714286 |
| ADCYAP1 | 0.01785714 | 0.44094488 | 95.5744681 | 1.33928571 | 0.02142857 |
| PFN2 | 47.1428571 | 206.173228 | 448.567376 | 219.357143 | 59.9285714 |
| STX1A | 4.07142857 | 74.4311024 | 164.964539 | 76.3214286 | 2.48571429 |
| ELMO1 | 0.19642857 | 15.4665354 | 194.184397 | 50.6071429 | 1.62142857 |
| ATP2A3 | 36.6071429 | 81.8326772 | 206.304965 | 122.714286 | 3.51428571 |
| PPT1 | 76.4107143 | 185.830709 | 332.93617 | 165.464286 | 111.228571 |
| PDX1 | 19.0535714 | 0.71062992 | 143.992908 | 52.0178571 | 57.65 |
| TMEM37 | 114.196429 | 16.5137795 | 181.51773 | 49.3571429 | 124.892857 |
| FAM105A | 7.98214286 | 28.9370079 | 151.787234 | 32.4107143 | 0.17857143 |
| PTEN | 51.4285714 | 57.6929134 | 217.439716 | 64.125 | 65.4928571 |
| MAFA | 0.01785714 | 0.14566929 | 29.8085106 | 0.71428571 | 0.00714286 |
| SH3GL2 | 2.57142857 | 61.4035433 | 121.510638 | 39.3928571 | 1.45714286 |
| CDKN1C | 4.75 | 26.261811 | 59.6666667 | 28.9821429 | 10.1428571 |
| PLCXD3 | 0.75 | 43.261811 | 120.120567 | 24.0178571 | 1.75714286 |
| TVP23B | 15.5714286 | 15.25 | 39.9929078 | 16.1964286 | 13.0142857 |
| DDIT3 | 33.8928571 | 27.1476378 | 199.553191 | 53.6428571 | 52.3071429 |
| ABHD10 | 19.8214286 | 23.9232283 | 93.9858156 | 51.0535714 | 17.8 |
| VPS37A | 22.125 | 33.8937008 | 71.858156 | 28.0892857 | 27.2714286 |
| RRAGD | 24.25 | 35.6673228 | 70.893617 | 19.9821429 | 23.1785714 |
| SURF2 | 27.1071429 | 31.8582677 | 58.751773 | 28.8035714 | 22.2642857 |
| RGS2 | 14.8928571 | 2.58858268 | 61.3829787 | 492.375 | 29.2 |
| SST | 25.5178571 | 510.427165 | 38.6808511 | 190088.375 | 13.6857143 |
| LEPR | 2.23214286 | 1.92125984 | 7.0141844 | 167.017857 | 2.55714286 |
| RBP4 | 2.48214286 | 17.1811024 | 2129.41844 | 4935.33929 | 10.4714286 |
| MS4A8 | 3.48214286 | 15.9153543 | 0.07801418 | 75.25 | 3.84285714 |

| | | | | | |
|---|---|---|---|---|---|
| UCP2 | 31.4107143 | 58.4173228 | 7.4822695 | 111.375 | 22.2571429 |
| SEC11C | 141.946429 | 216.423228 | 279.822695 | 886.178571 | 81.3071429 |
| UNC5B | 4.07142857 | 1.61220472 | 8.85815603 | 127.946429 | 6.43571429 |
| GABRB3 | 1.125 | 39.4212598 | 33.4255319 | 134.125 | 14.5214286 |
| BCHE | 0.03571429 | 0.50984252 | 0.09929078 | 70.3035714 | 0.00714286 |
| HEPACAM2 | 2.21428571 | 108.468504 | 165.716312 | 372.446429 | 1.00714286 |
| PCP4 | 0.41071429 | 66.5767717 | 158.70922 | 275.107143 | 1.94285714 |
| PCLO | 14.5178571 | 47.238189 | 54.1276596 | 121.678571 | 12.3357143 |
| BAIAP3 | 4.07142857 | 43.6299213 | 29.8368794 | 159.964286 | 5.76428571 |
| PRKACB | 6.03571429 | 17.2244094 | 19.9716312 | 69.7321429 | 15.1928571 |
| TENM3 | 4.10714286 | 12.6476378 | 10.6453901 | 82.625 | 0.00714286 |
| DHRS2 | 0.51785714 | 20.5531496 | 137.666667 | 253.053571 | 0.07142857 |
| DIRAS3 | 13.4642857 | 22.1141732 | 12.0425532 | 68.7321429 | 7.63571429 |
| LOC728392 | 4.10714286 | 33.003937 | 33.248227 | 87.8214286 | 0.80714286 |
| TPPP3 | 11.5535714 | 38.9724409 | 58.858156 | 154.446429 | 143.757143 |
| PKIB | 33.5 | 5.55905512 | 80.929078 | 112.910714 | 4.37142857 |
| GPC5-AS1 | 0.19642857 | 1.05511811 | 0.04964539 | 36.6785714 | 0.00714286 |
| PSIP1 | 10.5 | 9.38976378 | 15.8723404 | 57.8571429 | 15.4642857 |
| HHEX | 8.03571429 | 0.31692913 | 0.09219858 | 105.571429 | 67.2785714 |
| AKAP12 | 29.8035714 | 18.8031496 | 2.65957447 | 38.0714286 | 22.7785714 |
| LINC00643 | 0.30357143 | 26.0295276 | 38.5177305 | 66.5357143 | 0.58571429 |
| ARID5B | 22.5178571 | 19.3641732 | 41.4468085 | 44.3928571 | 24.2071429 |
| CLK1 | 33.2857143 | 36.0492126 | 64.3829787 | 72.8214286 | 51.0214286 |
| MAP9 | 26.4821429 | 23.8425197 | 19.7801418 | 43.9642857 | 20.6357143 |
| SYT4 | 0.85714286 | 40.8346457 | 64.2340426 | 77.2678571 | 0.88571429 |
| CBFB | 33.4642857 | 22.1299213 | 44.9574468 | 60.9821429 | 19.6214286 |
| CELF4 | 6.51785714 | 24.9940945 | 36.4468085 | 61.5892857 | 0.47857143 |
| KCNH2 | 1.30357143 | 25.5275591 | 33.6737589 | 51.4821429 | 3.07857143 |
| TINAGL1 | 50.6607143 | 1.49212598 | 1.05673759 | 0.05357143 | 618.8 |
| SPP1 | 564.857143 | 2.83267717 | 17.0567376 | 10.3035714 | 11419.3 |
| MMP7 | 165.071429 | 0.56496063 | 0.43971631 | 0.53571429 | 2184.17143 |
| AQP1 | 11.6785714 | 0.14566929 | 0.06382979 | 2.17857143 | 472.192857 |
| CFTR | 107.196429 | 0.27755906 | 0.19858156 | 0.21428571 | 1072.22143 |
| COL18A1 | 17.3035714 | 0.13582677 | 0.0212766 | 3.91071429 | 247.314286 |
| VTCN1 | 8.92857143 | 0.07480315 | 0.04964539 | 0.05357143 | 249.857143 |
| TSPAN8 | 127.178571 | 2.08464567 | 0.24822695 | 5.39285714 | 726.785714 |
| KRT19 | 598.982143 | 5.2007874 | 0.78723404 | 22.8035714 | 2437.67143 |
| KRT23 | 9.44642857 | 0.23622047 | 0.05673759 | 2.07142857 | 404.964286 |
| PMEPA1 | 64.4107143 | 0.21259843 | 2.11347518 | 5.76785714 | 469.635714 |
| ALDH1A3 | 2.94642857 | 0.07086614 | 0.04255319 | 0.03571429 | 282.271429 |
| PPAP2C | 53.7142857 | 0.15551181 | 0.17021277 | 0.08928571 | 280.728571 |
| SERPING1 | 402.017857 | 1.92913386 | 0.26241135 | 0.35714286 | 1324.08571 |
| SERPINA1 | 370.178571 | 1170.7874 | 4.31205674 | 913.214286 | 2714.00714 |
| TRPV6 | 39.25 | 0.03346457 | 0.0141844 | 0.03571429 | 158.792857 |
| PDLIM3 | 128.732143 | 0.1496063 | 0.95035461 | 14.1607143 | 472.364286 |
| CMTM7 | 15 | 4.2519685 | 12.3829787 | 1.05357143 | 132.678571 |
| LY6E | 17.6785714 | 28.6279528 | 13.5106383 | 12.9285714 | 210.735714 |

| | | | | | |
|---|---|---|---|---|---|
| HSD17B2 | 1.05357143 | 0.03937008 | 0.04255319 | 0.05357143 | 214.414286 |
| IGFBP7 | 43.8214286 | 176.175197 | 50.1560284 | 21.875 | 1112.42143 |
| SLC4A4 | 58.9821429 | 0.23818898 | 1.4822695 | 17.75 | 268.285714 |
| SERPINA5 | 341.267857 | 0.19488189 | 0.11347518 | 0.03571429 | 693.721429 |
| DEFB1 | 79.5714286 | 0.11023622 | 0.14184397 | 0.07142857 | 666.35 |
| ANXA3 | 84.25 | 2.96456693 | 9.18439716 | 0.96428571 | 287.95 |
| TFPI2 | 2.39285714 | 0.05314961 | 0.0141844 | 0.01785714 | 167.407143 |
| LGALS4 | 107.285714 | 0.07677165 | 0.0212766 | 0.03571429 | 302.9 |
| ANXA4 | 971.214286 | 42.7440945 | 4.55319149 | 20.4642857 | 1882.59286 |
| CTSH | 176.303571 | 8.71850394 | 3.24113475 | 1.75 | 596.807143 |
| PROM1 | 44.3035714 | 0.32874016 | 0.04255319 | 0.01785714 | 119.264286 |
| CALD1 | 53.6964286 | 1.12401575 | 18.7092199 | 38 | 198.4 |
| NRP1 | 9.83928571 | 17.4271654 | 2.61702128 | 15.6785714 | 160.214286 |
| SLC3A1 | 5.94642857 | 0.25590551 | 0.09929078 | 0.16071429 | 272.014286 |
| ANXA2P2 | 242.785714 | 33.4665354 | 26.4893617 | 2.35714286 | 704.857143 |
| ANXA2 | 487.571429 | 73.6102362 | 52.8652482 | 4.30357143 | 1369.9 |
| FLNA | 114.428571 | 3.77165354 | 1.4893617 | 2.25 | 309.814286 |
| WWTR1 | 46.3214286 | 0.52362205 | 0.87234043 | 1.14285714 | 122.8 |
| CLDN10 | 128.214286 | 0.15551181 | 0.09929078 | 6.39285714 | 427.392857 |
| CCND1 | 21.5892857 | 0.95866142 | 13.2340426 | 0.48214286 | 181.235714 |
| RBP1 | 224.482143 | 75.1358268 | 85.1205674 | 177.928571 | 603.678571 |
| HSPB8 | 61 | 0.03149606 | 0.18439716 | 6.96428571 | 159.9 |
| WFDC2 | 29.3392857 | 7.64370079 | 6.68085106 | 6.80357143 | 155.414286 |
| SDC1 | 54.0357143 | 1.44094488 | 0.0212766 | 3.01785714 | 176.735714 |
| PFKP | 58.5892857 | 76.3740157 | 35.2340426 | 44.2321429 | 202.071429 |
| CD9 | 103.535714 | 0.9507874 | 13.4184397 | 83.2857143 | 283.585714 |
| SLPI | 38.1964286 | 0.86220472 | 0.03546099 | 2.14285714 | 324.95 |
| GAS6 | 11.125 | 0.0984252 | 1.07092199 | 1.08928571 | 133.778571 |
| LITAF | 108.946429 | 1.8976378 | 5.07801418 | 25.2142857 | 343.778571 |
| FSTL1 | 6.53571429 | 5.76377953 | 0.09929078 | 0.17857143 | 118.95 |
| ONECUT2 | 25.9642857 | 2.02755906 | 7.43971631 | 0.46428571 | 102.364286 |
